# Quantitative constraints on flood variability in the rock record

**DOI:** 10.1038/s41467-023-38967-8

**Published:** 2023-06-08

**Authors:** Jonah S. McLeod, James Wood, Sinéad J. Lyster, Jeffery M. Valenza, Alan R. T. Spencer, Alexander C. Whittaker

**Affiliations:** 1 Department of Earth Science and Engineering, Imperial College London, London, SW7 2BX UK; 2 Department of Geosciences, The Pennsylvania State University, State College, PA 16801 USA; 3 Department of Geography, University of California, Santa Barbara, 1832 Ellison Hall, Santa Barbara, CA 93106 USA; 4 Science Group, The Natural History Museum, London, SW7 5HD UK

**Keywords:** Hydrology, Sedimentology, Hydrology, Geomorphology

## Abstract

Floods determine river behaviour in time and space. Yet quantitative measures of discharge variability from geological stratigraphy are sparse, even though they are critical to understand landscape sensitivity to past and future environmental change. Here we show how storm-driven river floods in the geologic past can be quantified, using Carboniferous stratigraphy as an exemplar. The geometries of dune cross-sets demonstrate that discharge-driven disequilibrium dynamics dominated fluvial deposition in the Pennant Formation of South Wales. Based on bedform preservation theory, we quantify dune turnover timescales and hence the magnitude and duration of flow variability, showing that rivers were perennial but prone to flashy floods lasting 4–16 h. This disequilibrium bedform preservation is consistent across 4 Ma of stratigraphy, and coincides with facies-based markers of flooding, such as mass-preservation of woody debris. We suggest that it is now possible to quantify climate-driven sedimentation events in the geologic past, and reconstruct discharge variability from the rock record on a uniquely short (daily) timescale, revealing a formation dominated by flashy floods in perennial rivers.

## Abstract (web summary)

Dunes and woody-debris preserved in the rock record have been used to quantify the magnitude and duration of flow events in ancient rivers, revealing a fluvial system dominated by flashy, storm-driven floods 300 million years ago.

## Introduction

Rivers are the most significant drivers of water and sediment transport across the continents^1^, and associated flood events play a key role in shaping landscapes, impacting ecosystems, and determining the magnitude, characteristics and locus of sedimentation on the surface of the Earth^2–10^. In principle, fluvial strata, which constitute a physical record of ancient river behaviour, provide a key archive to assess the impacts of flooding in the geologic past. An outstanding research challenge for geoscientists is to decode this archive effectively to evaluate: how, where and when fluvial deposits may record extreme events; the extent to which they can be quantified; and how much they may dominate the stratigraphic record^7,11–13^. This is particularly important as constraints on discharge variability from the geologic record provide a critical tool to understand past impacts of climate variability on river behaviour^8,14^. To-date qualitative insights into flow variability have largely been extracted from the rock record using facies analysis, including observations of supercritical flow indicators^10,15–19^. However, recent advances in our understanding of fluvial bedform dynamics in disequilibrium conditions raise the possibility of gaining quantitative insights into flow variability in ancient rivers^13,20^; when used together with sedimentary observations, these advances permit reconstruction of flood magnitudes and variability directly from fluvial stratigraphy.

**Fig. 1:**
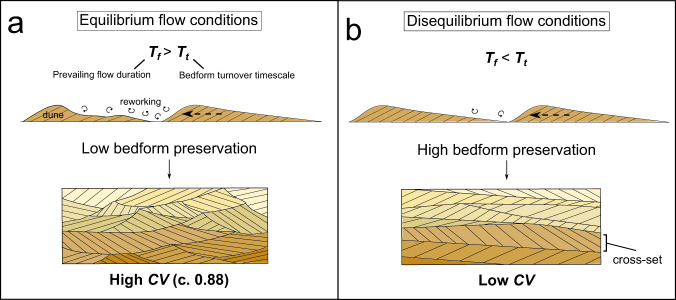
The hydrodynamic conditions that lead to differences in coefficient of variation of cross-set height, *CV*, recorded in cross-strata. **a** Dune migration and evolution in steady-state (equilibrium) flow conditions, and the resultant geometries of preserved cross-sets; **b** dune evolution and preservation in disequilibrium with prevailing flow, resulting in low *CV*.

The approach begins with the fundamental morphometrics of fluvial bedforms^20–28^, in particular dune-scale cross-strata, sub-critical bedforms which are ubiquitous in most ancient river deposits^20,21,24–26^. Cross-sets are preserved when dunes are not fully reworked by the prevailing flow, allowing the remaining bedform to become buried (Fig. 1). The “flood hypothesis” of bedform preservation^13^ states that enhanced bedform preservation occurs during floods (especially those with flashy hydrographs) when the formative flood duration, *T*_*f*_, is less than the timescale to rework a bedform, known as the turnover timescale, *T*_*t*_ (Fig. 1, see Table 1 for definitions). This is due to hysteresis in the adjustment of bedforms to changing flow conditions, meaning that when *T*_*f*_ < *T*_*t*_, bedforms do not have time to adjust in form to reach equilibrium with the prevailing flow. This key signal of flow variability can be extracted from dune-scale cross-strata using measurements of the distribution of heights (*h*_*xs*_) of preserved dune-scale cross-sets to calculate their coefficient of variation, *CV*^13^. In steady-state flow conditions, which may occur when *T*_*f*_ ≥ *T*_*t*_, the spread in cross-set heights in preserved stratigraphy is high: the *CV* is expected to be in the range 0.88 ± 0.3 because existing theory and experiments demonstrate that bedform migration across random bed topography with low angles of climb, in equilibrium with the prevailing flow, results in low bedform preservation and high *CV* (Fig. 1a)^13,21–23^. In contrast, when preservation occurs in disequilibrium conditions, which may arise due to flooding, the opposite is true (Fig. 1b). In this case, limited reworking of sediment within a dune results in lower *CV*^26^, with a greater proportion of the original dune preserved in stratigraphy. Disequilibrium bedform dynamics have been observed experimentally^29^, and recently dune cross-set *CV* has been used to indicate disequilibrium dynamics in stratigraphy^20,30^. However, flow variability is not the only origin of disequilibrium conditions: enhanced bedform preservation in disequilibrium conditions can also be caused by the presence of morphodynamic hierarchy, such as dunes migrating atop barforms^13,20,26^. Disequilibrium bedform dynamics caused by flow variability can therefore be difficult to definitively identify in the rock record, due to lack of independent evidence of variable discharge.

**Table 1:** Key palaeohydrological variables and definitions

Parameter	Definition		References
Mean cross-set height, ${h}_{xs}$	The mean from a distribution of heights measured within one cross-set.		
Original bedform height, *h*_*d*_	The original height of the bedform before preservation as a cross-set.		^21,22^
	$${h}_{d}=2.9\left(\pm 0.7\right){h}_{{xs}}$$		
Bedform preservation ratio, *h*_*xs*_/*h*_*d*_	The ratio of cross-set height to original bedform height, representing the proportion of the original height of the bedform preserved in the rock record.		^13,20^
Coefficient of variation of cross-set height, *CV*	The ratio of standard deviation to mean of cross-set height, measured along a single cross-set.		^21,22^
	${CV}=\frac{\sigma }{\mu }$	σ: standard deviation μ: mean	
Bedform turnover timescale, *T*_*t*_	The length of time taken for a bedform to be fully reworked by flow, or for the sediment in a dune to be displaced downstream by one bedform wavelength.		^12,13,70^
	${T}_{t}=\frac{\lambda {h}_{d}\beta }{{q}_{b}}$	λ: dune wavelength (≈7.3H) β: shape factor (≈0.55) ${q}_{b}$: unit bedload flux	
Prevailing flow duration, *T*_*f*_	The duration of the falling limb of the discharge event which generated the preserved bedform.		^12,13^
	${T}_{f}={T}_{t}T*$	$T*$: bedform disequilibrium number	
Flow intermittency factor, *I*_*f*_	The fraction of the total time in which bankfull flow would accomplish the same amount of water discharge as the real hydrograph.		^68^
	${I}_{f}=\frac{\Sigma Q\left(t\right)}{{Q}_{{bf}}\Sigma t}$	$\Sigma Q(t)$: sum of the time-dependent discharge *Q*_*bf*_: bankfull discharge $\Sigma t$: timespan	

Here, we test the flood hypothesis for enhanced bedform preservation in a location where unambiguous evidence of variable discharge conditions, including mass preservation of woody debris, can be combined with quantitative bedform and palaeohydrologic analyses. Therefore, we link for the first time bedform disequilibrium with stratigraphic evidence of flooding. In doing so, we demonstrate how sophisticated insights into water fluxes, climate and discharge variability can now be quantified for the geological past from stratigraphic data.

## Results and discussion

### Study area

**Fig. 2:**
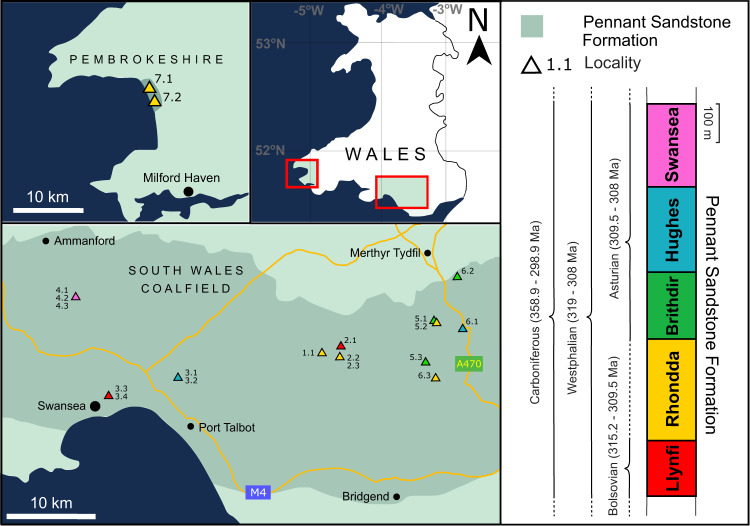
The South Wales and Pembrokeshire Coalfields, and the localities used for primary data collection. Pennant Formation geology is outlined after Jones and Hartley^35^. The stratigraphic column shows the five Members of the Pennant Formation, modified from Waters et al.^72^, and Barclay^73^ with age data from the British Geological Survey Geological Timechart. The localities are colour-coded by Member.

We focus on the Pennant Formation of South Wales, UK (Fig. 2), a 1.3 km thick succession of Upper Carboniferous (312.4–308 Ma, corresponding to the Moscovian age, or Bolsovian-Asturian substages) fluvial strata^31,32^. The five members of the formation (Llynfi, Rhondda, Brithdir, Hughes, Swansea) were deposited when South Wales was located near the equator, at a palaeolatitude of between 2.7^◦^N and 3.0^◦^S^33^. The formation is the product of rivers that drained the Variscan Mountains, flowing north-west^28^ across foreland basin floodplains^34,35^. The regional climate was warm and wet, with precipitation rates averaging 1.5–5 mm/day^33,36,37^. Individual catchment lengths and drainage areas reconstructed for multiple rivers in the Pennant system, based on outcrops in South Wales, average 130–200 km and ~4500−9500 km^2^, respectively^28,38,39^. Rapid sedimentation in a foreland basin setting (up to 340 m/Ma)^28,34^ resulted in a high-fidelity and high-temporal resolution record of fluvial processes across a c. 4 myr time period^31,34,35^.

The formation comprises bedded, channelised sandstone bodies, with well-preserved accretion sets and abundant dune-scale cross-bedding^28^. Separating the cliff-forming sandstone bodies are slope-forming fine-grained sediments representing floodplain deposition^35^. They contain abundant and well-documented coals^34,40^ indicating river migration across a forested, swampy foreland, characterised by high retention of surface water. As a result, the Pennant Formation has classically been divided into 3 main facies associations: fluvial channel, floodplain and mire^34,35^ (Supplementary Information 3). These characteristics are consistent with single-threaded or anastomosing rivers consisting of a few threads, which have been interpreted as showing perennial discharge regimes^15,28,31,34,35,40,41^. Qualitative observations of heterolithic deposits at channel margins (Supplementary Information 3) and the abundance of in-channel plant debris strongly point to the occurrence of flood events^31,34^, some of which entrained floodplain vegetation. These observations are consistent with the hypothesis in the thesis of Jones^34^, based on extensive facies analysis across South Wales, that the Pennant Formation contains evidence of variable discharge conditions. We therefore exploit this setting, including classical descriptions of facies associations^34,35^ as well as recent reconstructions of palaeo-rivers within the Pennant Formation^28^ to compare numerical and facies evidence of disequilibrium flow conditions related to floods, and in doing so, quantify discharge variability in a Carboniferous river system for the first time.

### Quantitative analysis of flood stratigraphy

We first consider whether this formation contains quantitative evidence of disequilibrium bedform preservation, consistent with the flood hypothesis^13^, and if so, what this implies about flood durations. We then place these results in the context of facies-based evidence of floods in the form of woody debris accumulations.

**Fig. 3:**
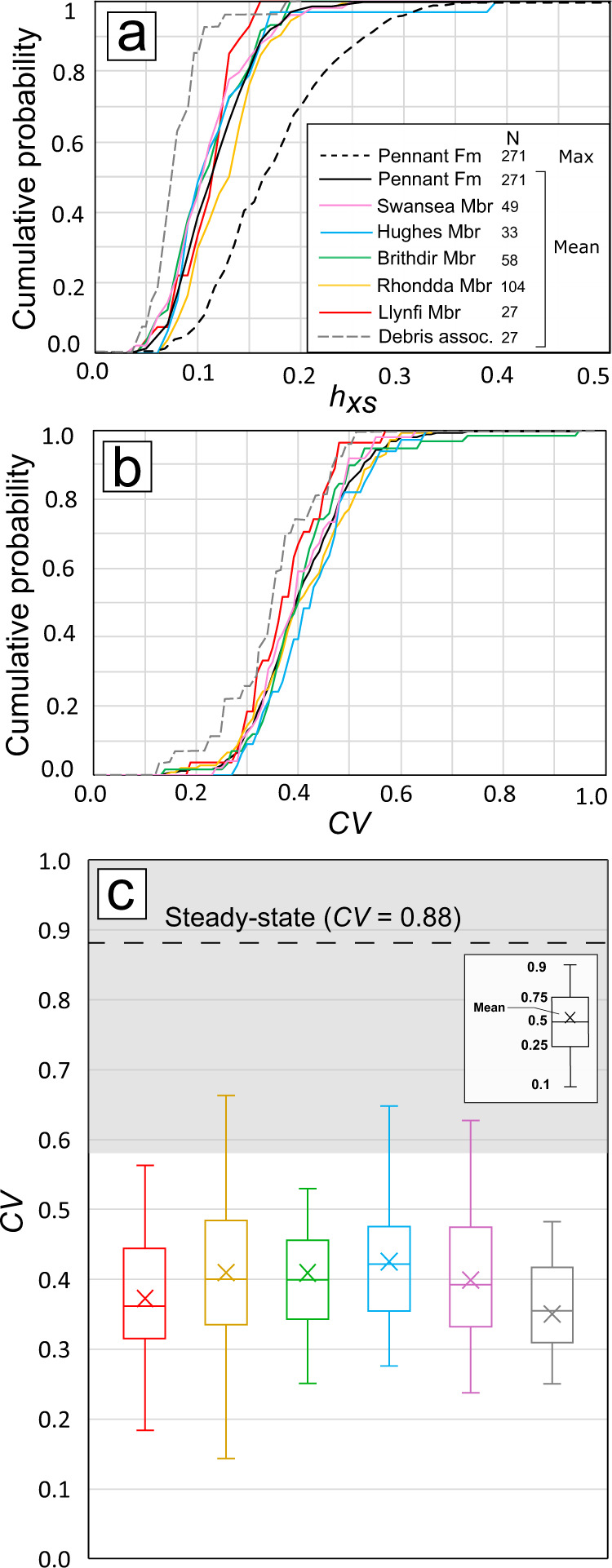
Cross-set data demonstrating disequilibrium bedform preservation. **a** Cumulative probability distributions of mean cross-set height for each member of the Pennant Formation, with distributions of the mean, 84th percentile, maximum for the Pennant Formation overall, and cross-sets associated with woody debris; **b** similar to (**a**), but with distributions of *CV;* **c** the *CV* of cross-set height for each member of the Pennant Formation. The dashed line and grey shaded region indicate the theoretical and empirical range of *CV* at steady state of 0.88 ± 0.3^21,22^, and the grey box represents cross-sets associated with woody debris.

The mean cross-set height, *h*_*xs*_, across the Pennant Formation was 0.12 m, with a median of 0.12 m and a standard deviation of 0.06 m (Fig. 3a). Values of maximum height measured within each cross-set average 0.19 m, and the median grain-size in the formation is 0.38 ± 0.06 mm (IQR), i.e., medium-grade sand. Two-tailed Kolmogorov–Smirnov (KS) tests show that the *h*_*xs*_ distributions of the Pennant Formation’s five Members are similar with 99.9% confidence (Supplementary Information S3h), and Fig. 3a shows that the distributions of mean *h*_*xs*_ follow a similar pattern across all members. This analysis indicates that measured samples of cross-sets have similar height distributions at member and formation level.

**Fig. 4:**
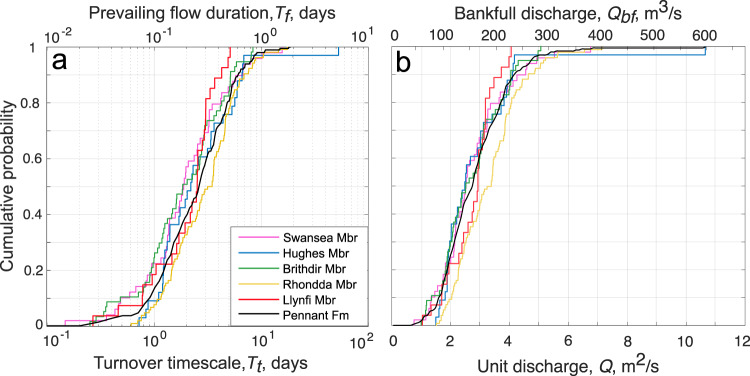
Cumulative probability distribution graphs showing key palaeohydrological variables. **a** The primary x-axis represents bedform turnover timescale, *T*_*t*_, in each member of the Pennant Formation, and the secondary x-axis indicates prevailing flow duration, *T*_*f*_, which we set as 0.1*T*_*t*_, following Leary and Ganti^13^; **b** the primary x-axis represents the unit discharge, *Q*, and the secondary x-axis represents the bankfull discharge, *Q*_*bf*_, calculated by multiplying *Q* by the average width of the channel, 55 m^28^.

Results also show statistically similar low *CV* distributions for all members with 90% confidence with median *CV* values spanning 0.36–0.42 (Fig. 3b). The median *CV* in the Pennant Formation is 0.40 and the mean is 0.41. We emphasize that these *CV* values are significantly lower than the theoretical value expected for steady-state bedform preservation of *CV* = 0.88 ± 0.3 (Fig. 4c)^13,21–23^. Indeed, 99.6% of cross-sets have *CV* below 0.88, and 96.7% have *CV* below 0.58 (0.88–0.3), suggesting that ~97% of dunes measured were preserved in disequilibrium with the prevailing flow at the time of deposition. These findings are consistent with theory and observations of disequilibrium (enhanced) bedform preservation^13,20,25,26^ (Fig. 3), and this signal of variable discharge conditions is consistent across all members of the Pennant Formation (Fig. 3c).

These data can be used to quantify bedform turnover timescales, *T*_*t*_, and prevailing flood durations, *T*_*f*_. We first explore what our data imply assuming a minimum theoretical bedform preservation ratio (*h*_*xs*_/*h*_*d*_, see Table 1) of 0.3^13,20,26^ to obtain estimates of the maximum durations of *T*_*f*_ and *T*_*t*_ (Fig. 4a). Then we evaluate the sensitivity of these results to higher bedform preservation ratios.

*T*_*t*_ calculations (Eq. 3) suggest dunes required a median of 3.2 days to be fully reworked by flow; similar results are recovered for all members of the Pennant Formation (Fig. 4a). Bedform theory and empirical observations^13^ demonstrate dunes preserved in the falling limbs of flashy floods, in disequilibrium with the prevailing flow, have a bedform disequilibrium number, T*, of <1, representing the ratio of *T*_*f*_ and *T*_*t*_. When the *CV* of cross-set height is as low as 0.4, as our calculations show, T* might be as low as 0.1^13,20^. This means given the average *T*_*t*_ of 3.2 days, *T*_*f*_ is reconstructed as ~8 hours (0.32 days). Flashy floods, which can be defined as having abrupt flow deceleration and T* ≪ 1^13^ are often associated with intense precipitation lasting less than half a day^42^ and can have almost symmetrical hydrographs^15^, so the total length of the average flood preserved in the Pennant Formation can be approximated as 16 h. To our knowledge this is the first time flood durations have been estimated for Carboniferous river systems. Based on paleohydrological calculations (Table 1 and ‘Methods’) we recover median bankfull discharge in individual channel threads as 140–160 m^3^/s, and considering previous reconstructions of several (i.e., 2–4) anastomosing threads^28^ this could be as high as 640 m^3^/s.

**Fig. 5:**
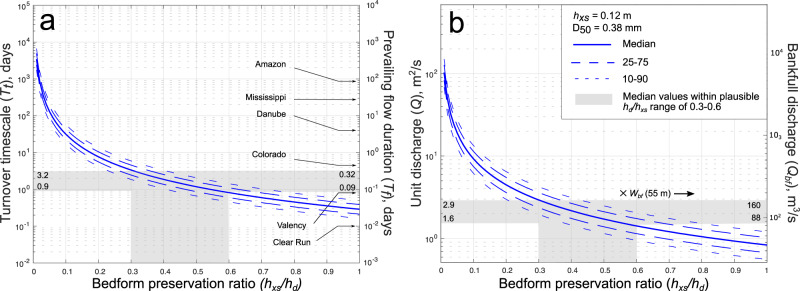
The effect of increased bedform preservation ratios on key palaeohydrologic parameters. **a** The primary y-axis indicates bedform turnover timescale, *T*_*t*_, and the secondary y-axis indicates prevailing flow duration, *T*_*f*_, when bedform disequilibrium number, *T*^∗^, is set as 0.1^13^, and *T*_*f*_ of 6 modern rivers are given for comparison (references in Supplementary Information); **b** the primary y-axis indicates unit discharge, *Q*, and the secondary y-axis indicates bankfull discharge, *Q*_*bf*_, when channel width is set as 55 m, the average for the Pennant Formation^28^.

Because disequilibrium (enhanced) bedform preservation due to flooding is indicated by our *CV* values (Fig. 3), the estimates presented in Fig. 4a are conservative maxima. The bedform preservation ratio, *h*_*xs*_/*h*_*d*_, is the ratio of measured mean cross-set height to estimated mean original dune height, and is influenced by the equilibrium dynamics of flow. Steady-state dynamics are implicit in many bedform scaling relations^22^, assuming *h*_*xs*_/*h*_*d*_ = 0.3, however plausible non-steady state values of *h*_*xs*_/*h*_*d*_ may be as great as 0.6, based on theory and experiments which show enhanced preservation during the falling limbs of flashy floods^13,20^. As *h*_*xs*_/*h*_*d*_ increases from 0.3 to 0.6 for a known *h*_*xs*_ (0.12 m on average for the Pennant Formation), the median *T*_*t*_ reduces from 3.2 days to 0.9 days (Fig. 5a). This means that while the falling limb of floods may be as long as 8 h assuming a ‘typical’ bedform preservation ratio of 0.3, *T*_*f*_ could be as short as 2 h assuming a bedform preservation ratio as large as 0.6. Durations are unlikely to be shorter than this as we do not see complete dunes preserved. The shaded regions in Fig. 5 illustrate the plausible range in palaeohydrologic parameters, with bankfull discharges for individual channels reconstructed from cross-set heights as between 88 and 160 m^3^/s (Fig. 5b). These could represent lower limits on bankfull discharge, with rare gravel-grade dunes suggesting discharges a factor of 1.5–2 greater than the sand fraction in the Pennant Formation^28^, although independent architectural constraints on channel morphology result in comparable discharge reconstructions, with a median of 140 m^3^/s per channel^28^.

Finally, we note that the flow intermittency factor of a river, *I*_*f*_, can be used to obtain quantitative contextual information on annual flow regime, and can be visualised as the proportion of the year a river would need to maintain bankfull discharge conditions to equal an estimate of the yearly water budget. For ancient fluvial systems such as the Pennant Formation, *I*_*f*_ can be estimated using published constraints on palaeogeographic and palaeo-precipitation rates (see ‘Methods’) to obtain a plausible annual water budget, and we exploit these to obtain first-order estimates of water flow intermittency factors for Pennant rivers. By comparing these constraints on mean annual discharge to our bankfull estimates (Fig. 4b), we estimate *I*_*f*_ = 0.17–0.44 (see ‘Methods’). This suggests that if the rivers of the Pennant Formation sustained bankfull conditions they could complete annual discharge in 62–160 days, which is consistent with perennial river systems, as discussed further below.

### Facies-based evidence for flooding

The quantitative analysis above, based on bedform theory, indicates that sediment deposition in the palaeo-rivers of the Variscan Foreland was controlled by disequilibrium bedform dynamics, which we relate to floods that had durations up to 16 h. But to what extent are these quantitative conclusions supported by facies-based observations? Fluvial channel facies in the Pennant Formation can be divided into three major lithofacies (*conglomerate, sandstone* and *heterolithic*) after Jones and Hartley^35^ which have been well-documented since the 1960s, and for which variable discharge conditions have been qualitatively suggested. We do not repeat these analyses but focus on the *conglomerate lithofacies*, first described by Jones and Hartley^35^ as conglomerates in which the clasts often comprise plant debris. We present new observations of woody debris, below, which we link to our quantitative approach. Further contextual details on facies that have been observed in the Pennant Formation^34^ are presented in Supplementary Information 3.

**Fig. 6:**
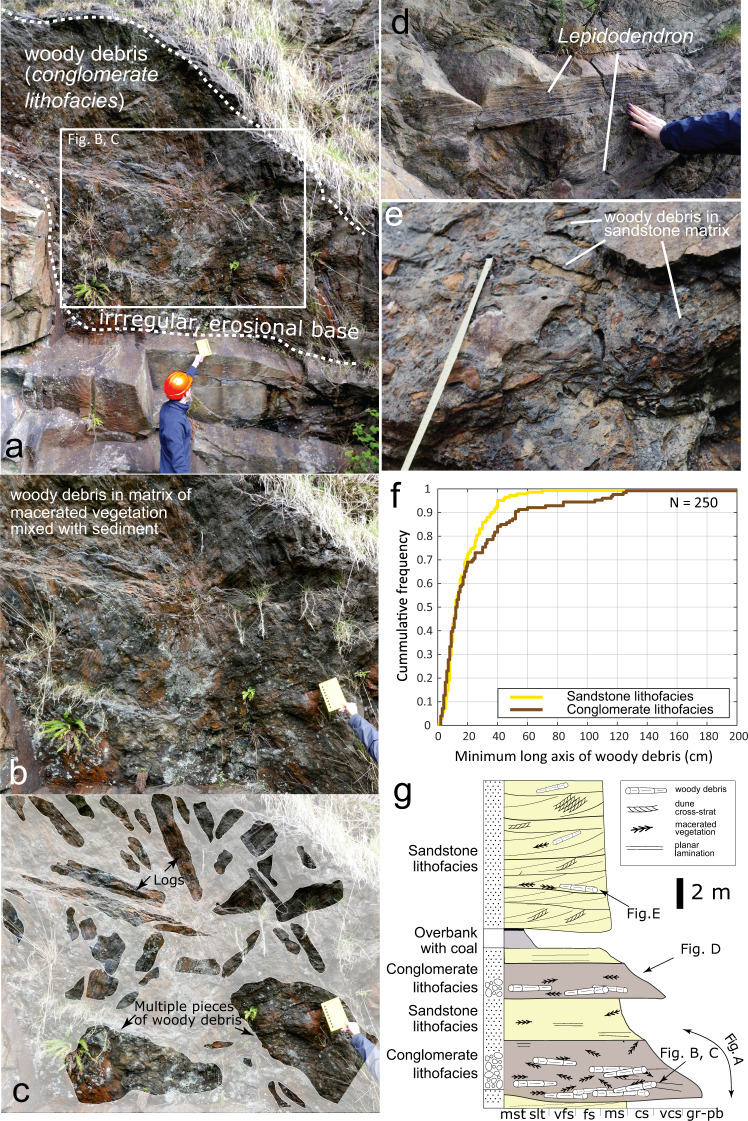
Examples of woody debris in the Pennant Formation, specifically in the Llynfi Member, at Kilvey Hill, Loc3.3. **a** The underside of the erosional base of a log-jam deposit in the *conglomerate lithofacies*, in which clasts comprise plant debris as opposed to rock fragments, overlying channel sandstone; **b**, **c** a closer view of this outcrop, with the largest woody debris fossils highlighted, noting that the matrix is composed of a mixture of sediment and macerated vegetation; **d** an example of well-preserved *Lepidodendron* fossils; **e** a debris bed in the *sandstone lithofacies*; **f** the cumulative frequency distribution of the minimum long axis of debris fossil found in the *sandstone* and *conglomerate lithofacies*; and **g** a schematic log displaying the typical features of the *conglomerate* and *sandstone lithofacies* in the Pennant Formation, using Kilvey Hill as an exemplar.

Dense accumulations of fossilised plant materials, or “plant conglomerates”^35^, are abundant and well-documented in the Pennant Formation. Plant fossils are preserved as a mixture of coalified compactions, compressions, as casts with well-preserved surface features, and occasional perimineralization. Identifiable fossils are mostly genus *Calamites* and *Lepidodendron*. *Calamites*, a genus of arborescent Equisetales (horsetails), grew in rapidly shifting and aggrading riparian settings^43^, proximal to channels, inhabiting levees, bars, and overhanging river channels. *Calamites* grew to its full height within 2 seasons, whereas *Lepidodendron* grew further from river channels, requiring more established substrate before reaching ~35 m in height and developing woody branches after 5–10 years of growth^31,41,44,45^. Although ubiquitous throughout the Pennant Formation, the densest plant accumulations (Fig. 6), historically referred to as “conglomerates”^35^ are observed in this study at 6 localities (Supplementary Information S3), but are documented throughout the formation^31,34,35,41,46^. They are characterised by large volumes of woody debris preserved at the bases of channel packages and accretion sets (Supplementary Fig. S2), only containing gravel-grade lithic clasts in a few rare instances. Conglomeratic debris beds are 0.25–3 m in thickness, and contain mostly *Lepidodendron* preserved as casts and compactions at varied stages of surface degradation. Fossils overlap and interlock, and occur in a matrix of highly macerated vegetation mixed with sand and organic-rich mud and silt. The *conglomerate lithofacies* contains a higher proportion of large debris fossils than the *sandstone lithofacies* (Fig. 6, Supplementary Information 3), and associated sediment is often poorly organised, but may contain a range of bedforms, from high-angle dune-scale cross stratification to upper plane-bed lamination. No in situ plant fossils (e.g., stumps) are observed.

The maximum length of woody debris we observed is 250 cm, with a median of 13 cm (Fig. 6f). The maximum reconstructed cylindrical volume of plant debris is 95,000 cm^3^ with a median of 237 cm^3^. While these woody debris accumulations have not before been linked with palaeohydrological observations, KS tests (see ‘Methods’ and S3, 4) demonstrate that dune cross-sets, where found in close association with woody debris in the *conglomerate lithofacies*, have an even lower *CV* than those documented elsewhere (Fig. 3) with 90% confidence. This shows that, whilst bedform preservation for sandy channel deposits is enhanced consistently at formation level, even greater enhancement is observed where debris-dominated facies associations are present. These data suggest that disequilibrium bedform preservation prevailed throughout the Pennant Formation and was particularly enhanced in flow associated with preservation of woody debris.

We interpret the observed debris conglomerates as log-jam deposits, generated by floods. First, the characteristics of the log-jam deposits observed here are similar to modern and ancient examples^10,16,46–49^, where debris orientation, sorting, and palaeobiology are comparable. Once plant material is in the river channel, log-jams can occur due to obstacles or flow separation between large objects such as bars or entire tree trunks^50^. Therefore, secondly, the formation of log-jams in the palaeo-rivers of the Variscan foreland is feasible due to the known presence of barforms and because *Lepidodendron* grew large enough to act as *key members* in log-jams^43^. Further, log-jams are known to have been frequent and diverse in Carboniferous rainforests^43^, and in ancient alluvial systems^4,10,16,49,50^. Moreover, The classic observations of Jones^34^ document woody debris up to 10 m long, suggesting the presence of material large enough to generate a significant obstruction in the channel^43^. We suggest, therefore, that the deposits observed represent transport jams as described by Gibling et al.^43^ and we link these events to the discharge variability documented using our quantitative bedform approach.

### Implications of bedform disequilibrium

Based on our quantitative bedform analysis, we document a *CV* of dune-scale cross-set height distributions in the Pennant Formation of 0.40 ± 0.07 (IQR) found throughout the unit (Fig. 3), which demonstrates that stratigraphy in the Pennant Formation preserves non-steady-state bedform dynamics. This is coupled with clear evidence for variable discharge conditions and the occurrence of floods. We show that 97% of observed cross-sets (*N* = 271) possess low *CV* (classified as ≤0.88 ± 0.3) consistent with enhanced dune preservation, and this appears to be the norm across up to 1.3 km of stratigraphy, a significant interval representing 4 Ma of deposition. Enhanced bedform preservation is being increasingly recognised in ancient fluvial systems^20,30^. Uniquely, our work in the Pennant Formation also links this signature to facies-based observations of flood-driven woody debris entrainment and deposition, and we interpret these disequilibrium conditions to likely reflect the prevailing flow during the falling limbs of floods. Based on bedform turnover timescale calculations, we reconstructed falling limb flood durations (*T*_*f*_) of 2–8 h, suggesting that relatively flashy floods had a total duration 4–16 h, with bankfull discharges of 140–160 m^3^/s per channel thread. This duration is consistent with published estimates of catchment size, with flow estimated to propagate through a catchment typical of the outcrops studied in 12–40 h^28^ (Supplementary Information 2). This is the first time that dune-scale bedform-based analyses of variable discharge conditions have been used to constrain flood durations in the ancient past. In conjunction with facies-based approaches, discussed below, this methodology provides a new way to quantify the magnitude and duration of floods in the stratigraphic record.

### Discussion of woody debris

We present evidence of log-jams and woody debris accumulations throughout the Pennant Formation, and we interpret these to have formed during floods, such as those that we quantify above. Rapid sedimentation and high-fidelity surface preservation of fossils in the *conglomerate lithofacies*, as well as their poor sorting and significant volume, speaks to high-magnitude storm-driven events. Plant accumulations including woody and peaty debris in accretion packages in other facies associations^35^ (e.g., the *sandstone lithofacies*, Supplementary Information 3) can also be explained by high-discharge events. These deposits are ubiquitous in the formation and occur in every member, implying deposition in rivers that were prone to discharge variability in a tropical ever-wet rainforest setting.

While plant material can be recruited into river channels by direct abscission, wind-blown input, and undercutting and collapse of the banks^51^, we suggest that the woody debris conglomerates present strong evidence of overbank flooding: firstly, the volume and density of many of the conglomeratic beds speak to the rapid recruitment of vegetation from large areas of forested floodplain, especially when considering estimates on Carboniferous tree spacing^52,53^. Secondly, the abundance of comminuted plant material gives insight into formation mechanism, implying maceration during transport, or prior decomposition on the forest floor. When found amongst large samples of woody debris this either requires flood water to transport rotted vegetation from the floodplain or to macerate fresh vegetation in high-energy flow. Further, these deposits are poorly sorted, with the lengths of measurable debris fossils in the 5−95% range being 0.03–1 m. It is unlikely this could be caused by gradual build-up of logs on/adjacent to a barform, and instead suggests rapid accumulation in a high energy setting. Third, the high quality of preservation of many fossils suggests rapid sedimentation, occurring predominantly during high and falling stages of flood events^50^.

Incremental floodplain cannibalisation is not favoured in this interpretation of log-jam debris recruitment not only due to the large volume of the deposits, but also due to the disproportionate absence of fossilised plant roots. If vegetation was recruited by bank collapse, this would place the entire tree, including roots, into the channel. However, these deposits do not contain roots, but mostly branches of *Lepidodendron*, which must have been collected by overbank flow where these organisms grew. *Lepidodendron* grew relatively far from river channels, requiring at least 5–10 years of stable growth before generating branches^51,54^, so it is unlikely that large volumes of branch material would have been recruited directly from the river bank. Furthermore, the absence of any in situ tree fossils suggests woody material was not sourced from plants living within the channel, consistent with palaeohydrologic reconstructions of these systems^28^ that show they were perennial. Palaeohydrological reconstructions show rivers channels were no wider than 200 m. Bank collapse on a scale large enough to incorporate enough of the floodplain into the channel to potentially cause a log-jam is therefore unlikely, and only occurs in the largest rivers today^55–58^. Even if undercutting and bank collapse were an additional mechanism, this process occurs especially during floods^43,50,59^.

Together, our quantitative analyses, coupled with our observations of log-jam deposits, show that disequilibrium conditions related to variable discharge and flooding are ubiquitous across 1.3 km of Welsh Carboniferous stratigraphy. Our data are unique in the ability to link qualitative facies indicators of potential discharge variability to quantitative evidence of enhanced bedform preservation. Where woody debris is found in the densest concentrations (i.e., log-jam deposits in the *conglomerate lithofacies* and plant-rich beds in the *sandstone lithofacies*), it coincides with lower cross-set *CV* to 90% confidence (Fig. 3b). Almost all cross-sets measured across the formation indicate disequilibrium preservation, interpreted to be driven by flashy floods, however, dunes associated with debris-transporting flood events are preserved with the lowest *CV* values. This demonstrates that debris accumulations record the same high-discharge events that are recorded by the disequilibrium preservation of dunes in ancient rivers, establishing dune cross-set *CV* as a robust indicator of discharge variability. This also highlights the critical importance of uniting facies-based evidence of variable discharge conditions with quantitative insights from bedform theory.

### Interpretation of discharge regimes

A number of indicators have been developed to identify systems with high discharge variability in the geologic record^10,15,16,60^, including Froude transcritical or supercritical structures and evidence of long periods free of discharge (e.g., in situ vegetation), often associated with strong seasonal precipitation patterns. However, facies evidence indicates that rivers had persistent discharge^61^ rather than strongly seasonal or highly intermittent discharge patterns^10,15,16,28,34,35^, consistent with our quantitative calculations. In the Pennant Formation transcritical- or supercritical sedimentary structures have been rarely observed, as sedimentation is dominated by subcritical dune bedforms alongside occasional upper plane-bed lamination in close association with woody debris. Moreover, supercritical conditions are not expected in these rivers given their reconstructed morphodynamics and flow velocities (see also Supplementary Information 3). The abundance and diversity of plants in upper Carboniferous coal forests implies that vegetation would colonise the river channel if long periods free of discharge occurred. However, no in situ vegetation has been observed in this formation, leading to the inference that rivers were perennial^15,16^. Moreover, the Pennant Formation’s fluvial channel facies contains abundant well-developed accretion sets, characteristic of perennial river deposits, as opposed to streams supplied largely by seasonal precipitation^15,17,18,62–65^. Serinaldi et al.^66^ also note that monsoonal regimes are typically characterized by sustained floods (5–25 days). *T*_*t*_ calculations, on the other hand, yield flood durations less than 1 day, which is inconsistent with models of subtropical systems, but consistent with flashy, precipitation- (storm-) driven floods in a perennial system. Further, Leary and Ganti^13^ found that sustained floods may have sufficiently long recession periods that bedforms reach equilibrium with the flow, in contrast with our results showing disequilibrium bedform preservation. All of these factors point towards a system not dominated by strong seasonality, but instead by storm precipitation on a daily timescale.

Finally, estimates of the water flux intermittency factor, *I*_*f*_, reconstructed for the Pennant Formation of 0.17 to 0.44 (see ‘Methods’) are not consistent with ephemeral discharge rivers^20,27^ but suggest the total annual water budget could be completed if bankfull conditions were sustained for around 1/3 of the year. The dominant grain-size, abundance of vegetation and perhumid climate is also potentially analogous to fluvial-dominated channels of the Mahakan Delta, Indonesia^67^. The intermittency factors we obtain are therefore broadly characteristic of perennial but variable flow in sand-bedded rivers^68^.

### Stratigraphic completeness

One final implication of the low *CV* values for fluvial cross-sets documented in this study is elevated bedform preservation ratios. The palaeohydrological and facies-based results of this study show the “unusual completeness”^11^ of the strata (in terms of bedform preservation) is likely due to discharge variability related to flooding^13,15,28^. This conclusion raises important questions about preservation of flow events in the stratigraphic record^11,21^. Variscan tectonics and associated accommodation generation undoubtedly contributed to the high rates of alluvial aggradation, as well as the preservation of woody debris^4^. However, given that almost the entire Pennant Formation contains the signature of disequilibrium bedform preservation, steady-state flow conditions appear to be disproportionately underrepresented. One explanation is that river sediment may behave in a state of disequilibrium more often than not due to the known hysteresis between flow conditions and adjusting dune morphology^69^. If this is true for the Pennant Formation, then this study offers further evidence that ancient rivers should not be treated as binary—either at steady-state or non-steady-state—but that disequilibrium bedform preservation is occurring regularly due to constant discharge variability.

However, given that we have extensive facies-based evidence for flood discharge conditions, our observations (e.g., Fig. 6) provide clear evidence for significant changes in flow conditions. Floods occurred over brief timescales, as we quantify above, therefore leaving perennial flow states to dominate the annual hydrograph, but evidently not the sedimentary record. In this scenario, the finding that 97% of observed cross-sets show *CV* values consistent with flood-driven discharge variability implies the exclusion of the vast majority of geologic time from the depositional record^11^. This study provides bedform-based evidence of disequilibrium flow conditions driven by flashy, storm-driven flooding, which we are able to link unambiguously with independent evidence of ancient floods for the first time, and adds to growing evidence that many systems may dominantly preserve sediment under conditions of bedform disequilibrium^20,29,30^. Consequently, we are able to reconstruct the signature of discharge variability on a daily timescale and our work illustrates how quantitative bedform analyses increasingly enable flood characteristics to be recovered from the rock record.

Taken together, these results demonstrate vividly how a careful combination of bedform and facies-based approaches can unlock fresh insights into Earth’s sedimentary systems and surface processes. This study represents the first quantitative investigation of bedform dynamics in Carboniferous palaeo-rivers and show how preserved bedforms can be used to extract signals of ancient discharge variability from fluvial stratigraphy. Palaeohydrological reconstructions reveal that the sand-bedded perennial palaeo-rivers in the Variscan foreland of the UK were significantly influenced by precipitation-driven flood variability, the signature of which dominated stratigraphy over a period of 4 Ma. Floods had duration 4–16 h, causing enhanced preservation of dunes and recruiting large volumes of woody debris, sometimes as log-jams, and floods had discharge magnitudes of 140–160 m^3^/s in individual channel threads.

## Methods

### Field observations

**Fig. 7:**
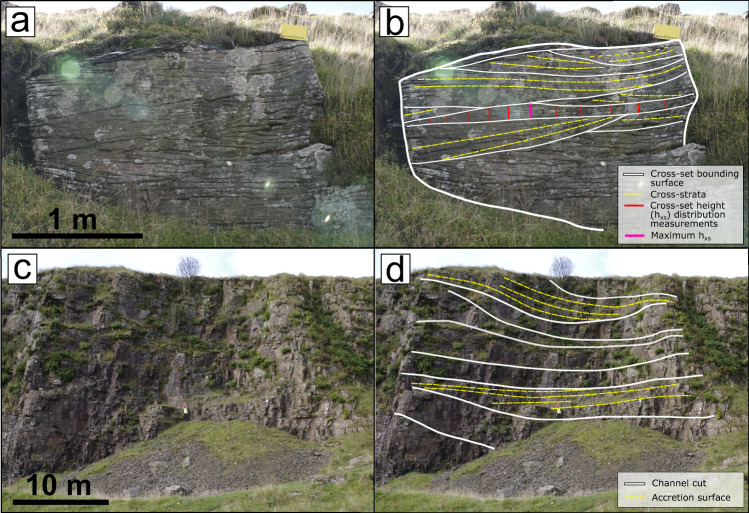
Field measurements at outcrop. **a**, **b** Methods of collecting cross-set height measurements, where the vertical bars make one cross-set height distribution, Locality 6.2; **c**, **d** architectural elements observed at outcrop scale, including accretion surfaces for use in Equation 7, Locality 2.1.

Primary data were collected in Autumn 2021 and Spring 2022 across 20 sites in the South Wales and Pembrokeshire Coalfields (Fig. 2; Supplementary Data 1, 2) from the five Members of the Pennant Formation. Primary data included cross-set height distributions (Fig. 7a, b), the geometries of various architectural elements (Fig. 7c, d), grain-size, and observations of flood facies (Fig. 6).

Cross-set height distributions were collected following the sampling strategy of Lyster et al.^20^, Ganti et al.^24^ and are explained in detail by Wood et al.^28^. Cross-set bounding surfaces were first identified, and cross-set height was measured (to a precision of ±5 mm) at regular intervals, with between 7 and 61 measurements per cross-set. We used cross-bed dip directions, palaeoflow estimates (both regional and local) and 3D outcrops with more than one exposed plane to ensure we sampled the cross-set parallel to the migration direction. A total of 4390 height measurements were taken across 271 cross-sets. Measurements of maximum cross-set height (with sample size *N* = 1735) were also collected separately. Relationships were established between the maximum and mean height from the recorded distributions (Supplementary Information 2), allowing estimation of mean *h*_*xs*_ from cross-sets where only the maximum value was measured. This increased the sample size of mean cross-set heights to *N* = 6125. For each observed cross-set, the grain-size of the sediment was also established (see Supplementary Information 2 for more detail on methods). The geometries of architectural elements, including the dimensions of channel and accretion packages, were measured using a Haglof Laser Geo laser range finder to a precision of ±5 cm. Data on woody debris fossils were collected by measuring their long and short axis to a precision of ±5 mm, and their location within the stratigraphic architecture was recorded.

### Quantitative palaeohydrology

Fundamental to the “flood hypothesis”^26^ is the detection of enhanced bedform preservation in fluvial strata. Measured *h*_*xs*_ distributions were used to calculate the coefficient of variation of cross-set height, *CV*, where:

1 $${CV}=\frac{\sigma }{\mu }$$

in which *σ* is the standard deviation and *μ* is the mean of the cross-set heights within a single cross-set. The *CV* reflects the preservation of the original dune, and therefore the equilibrium dynamics of flow: a *CV* of 0.88 is expected in equilibrium conditions^21–23^ and *CV* decreases as bedform preservation becomes enhanced (Fig. 1).

To calculate the original dune height from cross-sets observed in the field, the relationship established by Leclair and Bridge^22^ was used, based on previous theoretical work^21^:

2 $${h}_{d}=2.9\left(\pm 0.7\right){h}_{{xs}}$$

where *h*_*d*_ is the mean original dune height, and *h*_*xs*_ is the mean cross-set height. Values of *h*_*d*_ were then used in an array of further palaeohydrological calculations to build a complete picture of river morphodynamics. See Supplementary Information 2 for further detail on palaeohydrologic calculations and uncertainty.

To estimate uncertainty, Monte Carlo uncertainty propagation was used to generate a distribution of values for *h*_*d*_ that reflects the true spread of the data, following previous hydrological studies^20,27,70^. For Eq. 2, 10^6^ random samples were generated between bounds defined by *μ* − *σ* and *μ* + *σ* where *μ* is the mean and *σ* is one standard deviation. This was repeated for all formulae with a stated error, and propagated uncertainties were carried through.

Bedform turnover timescale (*T*_*t*_) is defined as the time to displace the volume per unit width of sediment in a bedform, i.e., the length of time required for a bedform to be completely reworked by the prevailing flow^12^. This parameter is used to indicate whether bedforms evolved in equilibrium with the prevailing flow, as a *T*_*t*_ that is greater than the duration of the prevailing flow, *T*_*f*_, implies a hysteresis that results in limited reworking of the bedform. This study determines *T*_*t*_ using the methods of Myrow et al.^12^ and Martin and Jerolmack^69^, in which:

3 $${T}_{t}=\frac{\lambda {h}_{d}\beta }{{q}_{b}}$$

where *λ* is dune wavelength (approximated as *λ* = 7.3*H*, where *H* is the formative flow depth), the shape factor *β* ≈ 0.55 and *q*_*b*_ is the unit bedload flux (See Supplementary Information 2). Myrow et al.^12^ define a dimensionless bedform disequilibrium number, *T*^*∗*^:

4 $$T*=\frac{{T}_{f}}{{T}_{t}}$$

Using data compiled from experiments and modern rivers by Leary and Ganti^13^, it is possible to establish plausible values of *T*^*∗*^ for calculated values of *CV*. Their results imply that dunes preserved in disequilibrium with falling-limb flood discharge lead to cross-sets low values of *CV* and *T*^*∗*^. Based on their data, we take 0.1 as a plausible value of *T*^*∗*^, meaning *T*_*f*_ = 0.1*T*_*t*_.

The flow intermittency factor, *I*_*f*_, is defined as the fraction of the total time in which bankfull flow would accomplish the same amount of water discharge as the real hydrograph^68^:

5 $${I}_{f}=\frac{\Sigma Q\left(t\right)}{{Q}_{{bf}}\Sigma t}$$

where Σ*Q(t)* is the sum of the time-dependent discharge, *Q*_*bf*_ is the discharge at bankfull conditions and Σt is the timespan. Flow intermittency requires estimation of a yearly water budget, and this necessitates a range of assumptions. Based on atmospheric general circulation models^33,36^, the palaeo-precipitation rate was estimated as between 1.5 and 2.5 mm/day, and catchment area has been estimated by Wood et al.^28^ as 4500−9500 km^2^, based on catchment scaling relationships^39^ and previously published palaeogeographic constraints^32^. Multiplying mean annual precipitation rates by the catchment area gives an estimate of the discharge (m^2^/s) supplied to the catchment, once modified to account for infiltration and evaporation of 20%^71^ (Supplementary Information 2).

### Statistical tests

Two-tailed Kolomogorov-Smirnov (KS) tests were performed in order to test the similarity of datasets, with the null hypothesis that the tested datasets have similar distributions. Firstly, the *h*_*xs*_ data collected in each member were tested against each other and against the data collected from the Pennant Formation as a whole. Secondly, the same tests were conducted for the cross-set *CV*. Finally, the *CV* values of cross-sets associated with woody debris were tested against those not associated with debris. See S2h, S2i and S2j in Supplementary Data 2, respectively, for these statistical tests.

## Supplementary information

Supplementary Information

Description of Additional Supplementary Files

Supplementary Data 1

Supplementary Data 2

## Data Availability

All data generated in this study are available in the Supplementary Information and have been deposited in the Figshare database [10.6084/m9.figshare.22564942, 10.6084/m9.figshare.22811333].
