# Supplementary material for: Quantitative constraints on flood variability in the rock record
Source: Nat Commun. 2023 Jun 8;14:3362. doi: 10.1038/s41467-023-38967-8 (PMC10250471; doi:10.1038/s41467-023-38967-8)
Supplement: Supplementary file 1 — Supplementary Information [file 41467_2023_38967_MOESM1_ESM.pdf]

Quantitative constraints on flood variability in the rock record

Jonah S. McLeod[1]*, James Wood[1], Sinéad J. Lyster[1,2], Jeffery M. Valenza[3], Alan R.T. Spencer[1,4], Alexander C. Whittaker[1].

[1]Department of Earth Science and Engineering, Imperial College London, UK, SW7 2BX.

[2] Department of Geosciences, The Pennsylvania State University, State College, Pennsylvania 16801, USA.

[3]Department of Geography, University of California, Santa Barbara, 1832 Ellison Hall, Santa Barbara, California 93106, USA.

[4]Science Group, The Natural History Museum, London, UK, SW7 5HD.

*jonah.mcleod18@imperial.ac.uk

Supplementary Information

Contents

Supplementary Data 1: Localities and access (.kmz)

Supplementary Data 2: Primary field data and statistical analysis

Supplementary Information 1: Table of localities

Supplementary Information 2: Extended methodology

Supplementary Information 3: Sedimentary facies

Supplementary Figure S1: Sedimentary logs of overbank deposits at Locality 4.3

Supplementary Figure S2: Fluvial facies photographs

Supplementary Data 1: Localities and access (.kmz)

A Google Earth .kmz file showing each locality, represented stratigraphy, and access information.

Supplementary Data 2: Primary field data and statistical analyses (.xlsx)

An excel workbook with all collected primary field data and statistical tests.

- S2a: Data log outlining collected datasets
- S2b: Cross-set height distributions
- S2c: Maximum cross-set height measurements
- S2d: Accretion and bedding measurements
- S2e: Package thickness measurements
- S2f: Woody debris measurements
- S2g: *CV* of cross-sets associated with woody debris
- S2h: Statistical test on cross-set heights between members
- S2i: Statistical test on *CV* between members
- S2j: Statistical test on *CV* between debris-associated and non-debris-associated cross-sets

Supplementary Information 1: Table of localities

Loc #	Loc name	Northing (BNG)	Easting (BNG)	Member	BGS Map Title	Access
**1.1**	Amphitheatre	5138.7	-00333.6	Rhondda	248: Pontypridd	A4107
**2.1**	Lower Bwlch Mountain Road	5138.7	-00331.94	Llynfi	248: Pontypridd	Bwlch-Y-Clawdd Road
**2.2**	Upper Bwlch Mountain Road	5138.55	-00332.12	Rhondda	248: Pontypridd	Bwlch-Y-Clawdd Road
**2.3**	Welcome to the Valleys Sign	5138.4	-00332.09	Rhondda	248: Pontypridd	Bwlch-Y-Clawdd Road
**3.1**	Top of Disused Mineral Railway	5138.52	-00347.71	Hughes	247: Swansea	The Incline - Briton Ferry Woods Car Park
**3.2**	Below Mountain Coal - Disused Mineral Railway	5138.46	-00348.06	Hughes	247: Swansea	The Incline - Briton Ferry Woods Car Park
**3.3**	Kilvey Hill West	5137.60	-00354.91	Llynfi	247: Swansea	Path off Harbour View Road
**3.4**	Kilvey Hill East	5137.60	-00354.80	Llynfi	247: Swansea	Path off Harbour View Road
**4.1**	Darren Serth Quarry: First Storey	5143.73	-00356.95	Swansea	230: Ammanford	Lliw Reservoir Car Park

**4.2**	Darren Serth Quarry: Round Corner	5143.78	-00356.86	Swansea	230: Ammanford	Lliw Reservoir Car Park
**4.3**	Darren Serth Quarry: Second Storey	5143.72	-00356.95	Swansea	230: Ammanford	Lliw Reservoir Car Park
**5.1**	Llanwonno Road Quarry	5140.24	-00322.43	Brithdir	248: Pontypridd	Llanwonno Road
**5.2**	Llanwonno Road	5140.24	-00322.43	Brithdir	248: Pontypridd	Llanwonno Road
**5.3**	Quarry Above Porth	5137.56	-00323.96	Brithdir	248: Pontypridd	Layby off Graigwen Road
**6.1**	Above Abercynon	5139.47	-00319.88	Hughes	248: Pontypridd	Layby on unnamed road off Goitre-Coed Road
**6.2**	Mynydd Cilfach-yr-encil	5142.75	-00319.79	Brithdir	231: Merthyr Tydfil	Dowlais Road
**6.3**	Bridge Street	5136.71	-00323.034	Rhondda	248: Pontypridd	Bridge Street
**7.1**	Nolton Haven North Cliff	5149.43	-00506.65	Rhondda	226/227: Milford	Nolton Haven Car Park
**7.2**	Maidenhall Point	5150.58	-00506.99	Rhondda	226/227: Milford	Pebbles Café Car Park
**7.3**	Watchman's Hut	5141.12	-00333.20	Rhondda	248: Pontypridd	Rhigos Road

Supplementary Table 1: The localities used for primary data collection and their locations.

Supplementary Information 2: Extended methodology

Scaling relationships obtained in order to extract median cross-set height from measurements of maximum cross-set height were as follows:

**Member**	**Mean/Max**	**N**
Combined (Pennant)	0.626	271
Swansea	0.625	49
Hughes	0.596	33
Brithdir	0.636	58
Rhondda	0.618	108
Llynfi	0.656	27

Supplementary Table 2: The derived scaling ratios between mean and maximum cross-set height for each Member of the Pennant Formation, including the number, N, of cross-set height distributions obtained from each Member.

There are a number of empirical relationships used in our numerical analyses in order to reconstruct palaeohydrology in the rivers of the Variscan Foreland. Eq. 2 in the main text,

$$h_d = 2.9(\pm 0.7)h_{xs}$$

was formulated by Leclair and Bridge[1] and was based on previous theoretical work (e.g. [2–4]). Uncertainty is due to natural variability in bedform preservation ratio. This uncertainty is important to incorporate in palaeohydrologic reconstruction, so Figure 5 (main text) illustrates how this manifests in estimations of bedform and flow dynamics. The main assumption contained within this relation is that the mean cross set height, h_{xs}, is measured correctly, i.e., from a distribution of closely spaced vertical profiles. Eqs. 3 and 4 in the main text,

$$T_t = \frac{\lambda h_d \beta}{q_b}$$

$$T* = \frac{T_f}{T_t}$$

also contain implicit assumptions regarding bedform shape and flow style, but these are well-established in bedform research and have been relevant for decades in studying the dynamics of bedform adjustment[5–8]. The T_f of modern rivers shown in Figure 5 in the main text provide context for the discharge regimes typical of the Variscan Foreland, and these values were sourced from the following publications: [9–14]

Formative flow depth, H, was calculated using the relationship established by Bradley and Venditti:[15]

$$H = 6.7h_d$$

(Eq. 6)

where the value of 6.7 is an approximation of a scalar range with 50% probability between 4.4 and 10.1. This relation is derived from compiled field data, and could also be set as $h = 6.96H^{0.95}$.

Primary grain-size data were necessary to establish palaeoflow conditions. Most cross-sets were preserved in sand-grade deposits, but we also observed rare pebble-grade cross-sets. The grain-size of sand-fractions (<2 mm) was estimated in the field according to size terms of the Wentworth[16] classification, confirmed by processing of grain-size images in ImageJ software, and the median grain-size (D_{50}) was extracted. These data were used to calculate palaeoslope using the method of Trampush et al[17] where:

$$logS = a_0 + a_1 logD_{50} + a_2 logH$$

(Eq. 6)

in which S is channel slope, D_{50} is the median grain-size, and constants $a_0 = -2.08 \pm 0.036$, $a_1 = 0.254 \pm 0.016$, and $a_2 = -1.09 \pm 0.044$. This relation was used as it is most appropriate for the range of grain sizes observed in this study, and it is consistent with the bedload flux model used[18]. To propagate the errors included in the constants, 10^6 values were generated of each (Monte Carlo uncertainty propagation).

Channel width is an important metric due to its necessity in establishing a total discharge, as opposed to discharge per unit width. This permits quantification of discharge during bankfull events, which must be known in order to estimate flood capacities. However, channel width is difficult to constrain from outcrop. The method established by Greenberg et al.[19] describe the widths of individual river threads, and was used due to higher sampling potential than direct measurements of outcrop width. Furthermore, outcrop width indicates the maximum width of the channel belt, rather than individual river channels, the latter of which has greater utility in palaeohydrologic reconstructions. In the method of Greenberg et al.,[19] lateral accretion package widths are used to estimate the total width of the channel:

$$W_{bf} = (2.34 \pm 0.13)W_{bar}$$

(Eq. 7)

where W_{bf} is bankfull width, and W_{bar} is the width of a bar package, defined as the distance between the locations that mark the 95% values of the asymptotes formed by the lateral accretion package. Exemplar asymptotic bar packages are shown in Figure 4d. Width estimates were made in tandem with estimates of planform morphology and fluvial style[20,21]

as it is implicit in the Greenberg et al[19] method that rivers are single-threaded. The total width of outcrops, measured in Google Earth, was used to indicate an upper limit on the width of the total channel belt.

Flow velocity was calculated using Manning's equation:

$$U = \frac{1}{n} H^{\frac{2}{3}} S^{\frac{1}{2}}$$

(Eq. 8)

where n is Manning's constant, set as 0.03.[22] In this well-established methodology, water discharges were then estimated using $Q = UH$ to obtain discharge per unit width (i.e., unit discharge, Q), and channel width, W_{bf}, was estimated (Eq. 7), to obtain bankfull discharge ($Q_{bf} = U\, H\, W_{bf}$).

Unit bedload flux, qb, was estimated using the methods of Mahon and McElroy[18]:

$$q_b = (1 - \phi)\frac{h_d V_c}{2}$$

(Eq. 9)

$$logV_c = \beta_0 + \beta_1 logS$$

(Eq. 10)

where Vc is the bedform migration velocity, β_0 and β_1 are constants ($\beta_0 = 0.6113 \pm 0.144$, $\beta_1 = 1.305 \pm 0.0515$), and φ is a dimensionless bed porosity of 0.5.[18] These formulae were derived based on field and experimental data, and implicit in this model is the bedform height relation of Leclair and Bridge[1].

The flow intermittency factor, I_f, is defined as the fraction of the total time in which bankfull flow would accomplish the same amount of water discharge as the real hydrograph[23] (Eq. 5 in the main text). This metric is important for analysis of fluvial discharge variability. Flow intermittency requires estimating a yearly water budget, and this necessitates a range of assumptions. Based on atmospheric general circulation models[24,25] the palaeo-precipitation

rate was estimated as between 1.5 and 2.5 mm/day (0.55 - 0.91 m/yr), and catchment area has been estimated as 4500 - 9500 km^2 by Wood et al.[26] From these assumptions we have estimated the expected mean annual discharge, Q_a, for a given channel:

$$Q_a = Q_{wb}A$$

(Eq. 11)

where A is the catchment area, and Q_{wb} is the water budget, representing the water that enters the catchment as precipitation, assuming a 20% loss to infiltration and evaporation.[27] Reconstructed catchment lengths of 135 – 200 km and average flow velocities were used to estimate flood propagation timescales through the catchment. Although uncertainty is expectedly high due to uncertainty in palaeogeographic reconstruction, catchment shape and size, and precipitation magnitude and distribution, flood propagation could have occurred on similar timescales to reported flood durations.

Supplementary Information 3: Sedimentary Facies

The sedimentary facies within the Pennant Formation have been well-documented in classic literature since the 1960s. Rather than re-stating the observations of Jones[28] and many other authors[29–33] we focus in the main text on the facies indicators of variable discharge, and provide here some more detailed context. The fluvial successions of the South Wales Coalfield are recognised in literature since at least the 1920s[34] with previous mapping work completed by the British Geological Survey from the early-mid 1800s. In the 1940s and 50s, significant advances in description and classification of Welsh Carboniferous stratigraphy were driven by interest in the Coal Measures by authors such as Moore[35,36] (1945, 1948) and Blundell[37] (1952). By the 1960s, sedimentary geology in South Wales was a topic of great interest in the geoscientific community, headed by Kelling[33,38] (1964, 1968). In 1977, the PhD thesis of Jones (C. M.)[28] documented in impressive detail the fluvio-deltaic sequences of the Pennant Sandstone, laying the groundwork for later investigations of tectono-stratigraphy and palaeohydrology by Jones (J. A.) and co-authors[29,39]. Jones and Hartley[29] described three main facies present in the Pennant Formation: fluvial channel, floodplain, and mire. Within the fluvial channel facies, there are three distinct lithofacies: *conglomerate*, *sandstone*, and *heterolithic*. In particular, Figures 2, 3 and 6 of Jones and Hartley[29] illustrate these. Below,

we outline observations of each of these facies, before describing in detail the facies evidence for discharge variability.

S5.1 Observations

Sandstone lithofacies

This facies is the most volumetrically significant in the formation. It contains large channel sandstone packages and well-developed lateral and downstream accretion, with abundant dune-scale cross-bedding. This facies represents anastomosing channel belts 100 – 200 m wide and 2 – 3 m deep, transporting medium-grade sand on average[26]. As recognised by previous stratigraphic and palaeobotanical work[26,28,31,40], plant fossils are ubiquitous throughout the formation, occurring mostly between accretion packages and on accretion surfaces. Fossils are generally isolated, i.e., not in contact with other fossils, and they are observed both near the bases and tops of barforms. Plant fossils are preserved as a mixture of coalified compactions, compressions, as casts with well-preserved surface features, and occasional perimineralization. Identifiable fossils are mostly genus *Calamites* and *Lepidodendron*, and rarer *Stigmaria*. *Calamites* is a genus of arborescent Equisetales (horsetails) extent through the Carboniferous until the Mid-Permian, which grew in rapidly shifting and aggrading riparian settings[41], proximal to channels, often inhabiting levees, bars, and overhanging river channels. *Calamites* grew to its full height within 2 seasons, whereas *Lepidodendron* grew further from river channels, requiring more established substrate before reaching 35 m in height and developing woody branches in 5 – 10 years.[31,40,42,43] Also observed are lenses of compressed, macerated vegetative material, mostly coalified. The median length of woody debris fossil is 12.5 cm and the median width is 5 cm. The largest sample observed was 250 x 40 cm. On average, the exposed area of fossils is 56.5 cm^2 and the median reconstructed cylindrical volume is 221 cm^3.

Conglomerate lithofacies

Conglomerates are well-documented in the Pennant Formation, and notably, dense accumulations of plant material are abundant through space and time. The densest plant accumulations, or “conglomerates” are observed in this study at 6 localities throughout the Pennant Formation, but are documented throughout the formation. They are characterised by large volumes of woody debris preserved at the bases of channel packages and accretion sets. Debris conglomerates are 0.25 – 3 m in thickness, and contain mostly *Lepidodendron*

preserved as casts and compactions at varied stages of surface degradation. Fossils overlap and interlock with no preferred orientation, and occur in a matrix of highly macerated vegetation mixed with sand, and organic-rich mud and silt. The debris fossils within conglomeratic beds contain a higher proportion of large samples than in the *sandstone lithofacies*, and associated sediment is poorly organised, but may contain a range of bedforms, from high-angle dune-scale cross stratification to UPB lamination. No in-situ plant fossils (e.g. stumps) are observed. The median size of fossils is 12.5 x 4 cm, with a maximum of 250 x 22 cm. The median area of fossils is 63 cm^2, with a reconstructed cylindrical volume of 237 cm^3.

Heterolithic lithofacies

Heterolithic successions occur on a range of spatial scales and are also well-documented in the formation. They comprise interbedded units of fine sandstone, silts and muds, and may occur on the margins of major channels (See S6).

S5.2 Interpretations

Our observations of the *conglomerate lithofacies* are consistent with those of previous studies, although Jones[28] observed logs up to 10 m long. Whilst well-documented, woody-debris accumulations have not before been linked with palaeohydrology in this formation.

We interpret the observed debris conglomerates as log-jam deposits based on the following reasoning. Firstly, log-jams are known to have been be frequent and diverse in Carboniferous rainforests,[41] and are well-studied occurrences in ancient alluvial systems[41,44–48] . Once plant material is recruited to the river channel, log-jams can occur due to obstacles or flow separation between large objects such as bars or entire tree trunks.[46] Therefore, secondly, the formation of log-jams in the palaeo-rivers of the Variscan foreland is feasible due to the known presence of barforms and because Cordaitaleans grew large enough to act as key members in log-jams.[41] No fossils were observed on the scale of channel widths reconstructed by Wood et al.,[26] but Gibling et al.,[41] state that, in any case, key members are unlikely to be exposed in stratigraphy. Third, the characteristics of the log-jam deposits observed here are also similar to modern and ancient examples,[44,45,47,49–51] where debris orientation, character, and palaeobiology are comparable, and based on this, we suggest that the deposits observed represent transport jams as described by Gibling et al.[41]

Ferguson[52] states that plant material can be recruited into river channels by direct abscission, wind-blown input, undercutting and collapse of the banks, and flooding. Based on the justifications below, we suggest that these debris conglomerates present strong evidence of flooding. Firstly, the volume and density of many of the conglomeratic beds speak to the rapid recruitment of vegetation from large areas of forested floodplain, especially when considering estimates on Carboniferous tree spacing[53,54]. Secondly, the abundance of comminuted plant material gives insight into formation mechanism, implying maceration during transport, or prior decomposition on the forest floor. Either way, when found amongst large samples of woody debris (thus discounting wind-blown recruitment), this either requires flood water to transport rotted vegetation from the floodplain or to macerate fresh vegetation in high-energy flow. Furthermore, these deposits are poorly sorted, with the lengths of measurable debris fossils in the 5 - 95% range being 3 – 100 cm. This is unlikely explained by gradual build-up of logs due to a barform, and instead suggests rapid deposition in a high energy setting. Third, the quality of preservation of many fossils suggests rapid sedimentation, occurring during high and falling stages of flood events[46]. Our rapid reconstructed flood recession timescales also imply short transport distances, supporting observations of well-preserved leaf-cushions on some samples within log-jam deposits.

Incremental floodplain cannibalisation is not favoured in this interpretation of log-jam debris recruitment not only due to the large volumes of the deposits, but also due to the disproportionate absence of preserved roots. If vegetation was recruited by bank collapse, this would place the entire tree, including roots, into the channel. However, these deposits contain mostly branches of *Lepidodendron*, which must have been collected by overbank flow where these trees grew. Furthermore, the absence of any in-situ tree fossils suggests woody material was not sourced from plants living within the channel, consistent with palaeohydrologic reconstructions of these systems[26]. Even major bank collapse events likely could not explain the observed deposits: palaeobotanical reconstructions show rivers in these environments did not have tall, steep banks, and collapse on scales large enough to potentially cause log-jams are only common in the largest rivers, such as the Brahmaputra and Mississippi[55–58]. The palaeo-rivers of the Pennant Formation were a maximum of 200 m wide[26], and this limits the area from which vegetation could be recruited by bank collapse. *Lepidodendron* also grew relatively far from river channels, requiring at least 5-10 years of stable growth before generating branches[59,60] , so branch material could not be recruited directly from the river bank. Even if undercutting and bank collapse were the preferred mechanism, this process

occurs especially during floods[41,61,62], reinforcing our preferred explanation for these log-jam deposits: flooding.

The woody debris observed in the *sandstone lithofacies* may have been recruited during flooding as explained above, but their composition, containing a higher proportion of *Calamites*, opens the possibility of recruitment by gradual bank cannibalisation, since they grew much faster, and closer to the river channel. Nevertheless, the high quality of fossil preservation suggests rapid sedimentation, and since bank undercutting occurs preferentially during floods, their ubiquity in the sandstone facies throughout the formation suggests discharge variability contributed to the debris observed. Compressed and fragmented lenses of macerated, coalified plant material observed within these deposits also indicate overbank flow which may have placed rotting vegetation from the floodplain into the channel.

Finally, we interpret the *heterolithic lithofacies* observed as representing overbank successions or crevasse splay deposits. These deposits indicate that discharge intermittently reached at least the level of the floodplain, and crevasse splays are also known to occur during significant flood events[63–65] so their recognition in the Pennant Formation here and by previous authors (e.g. [28]) may indicate flow variability.

The sedimentary structures observed across the formation are Froude sub-critical, from ripples to upper plane-bed lamination. However, the absence of transcritical or supercritical bedforms does not refute interpretations of flood-influenced deposition. In general, preservation of Froude supercritical structures is not expected in perennial systems, except potentially atop barforms during waning flow. They are much more commonly preserved in ancient ephemeral systems driven by seasonal precipitation. Further, in order for the reconstructed Froude number to rise 1, permitting formation of transcritical bedforms, either the flow velocity must increase roughly four-fold from the average to 4.75 m/s, or the flow depth must decrease to 0.16 m, less than half the height of the average dune. These values are not consistent with morphodynamic interpretations, especially considering the lack of facies evidence for common supercritical flow.

Supplementary Figure S1: Sedimentary logs of overbank deposits at Locality 4.3

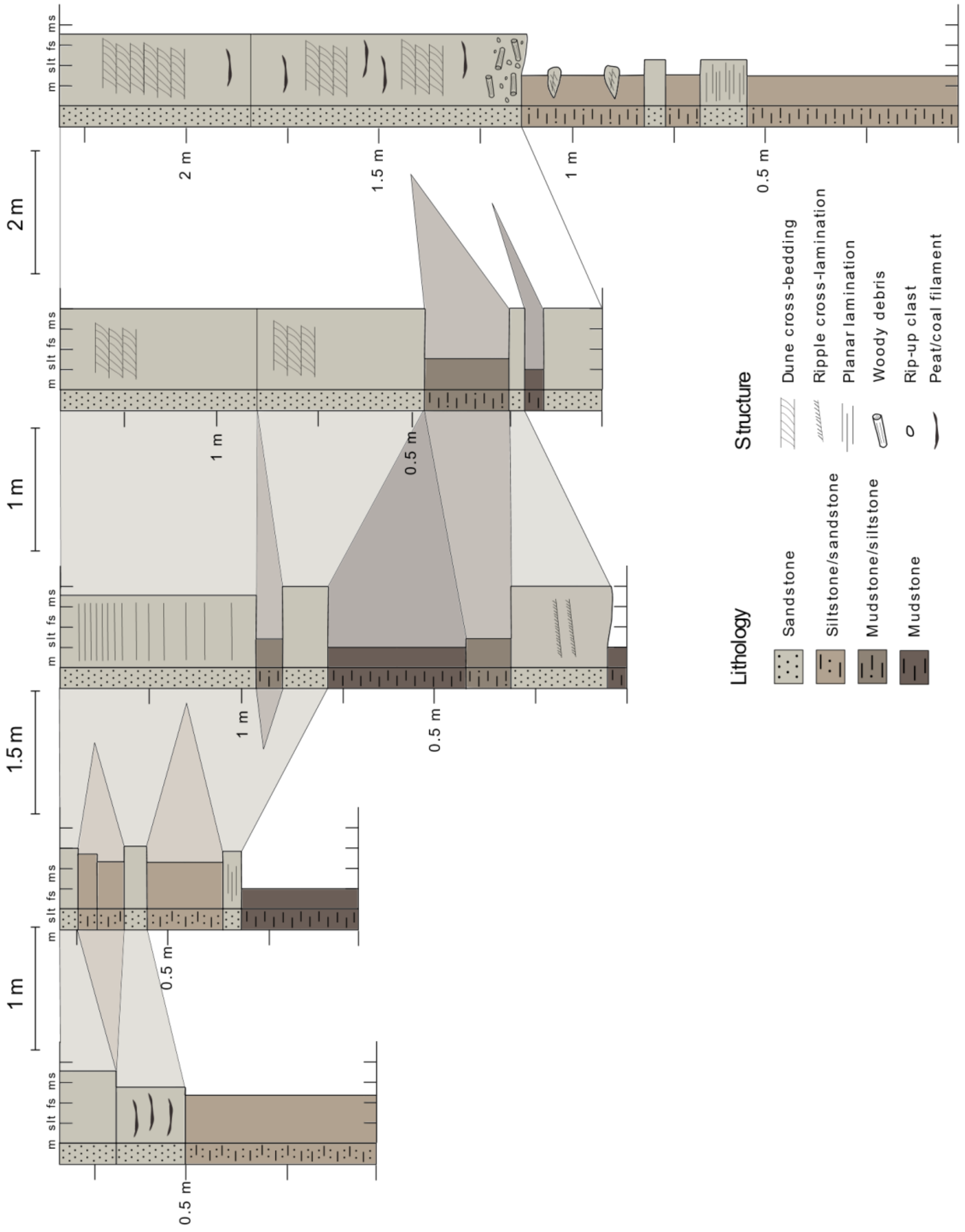

Supplementary Figure S2: Fluvial facies photographs

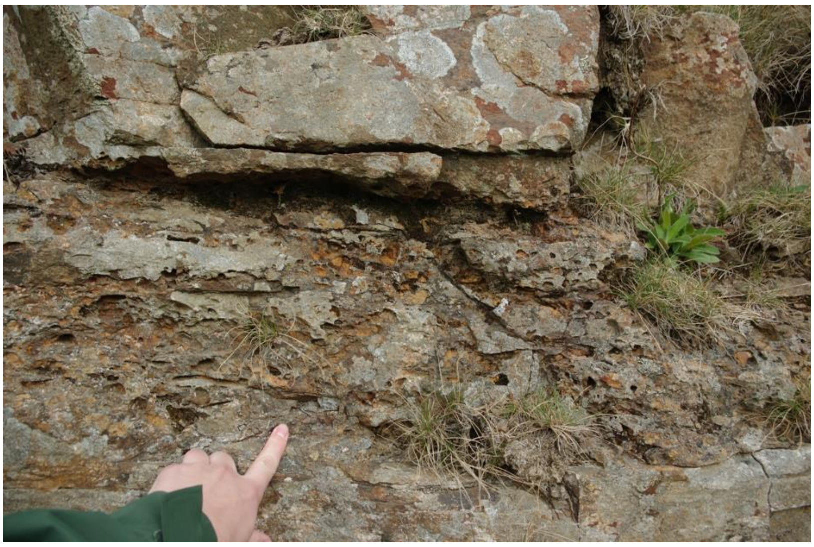

Supplementary Figure S2.1. Conglomeratic bed at Loc2.2

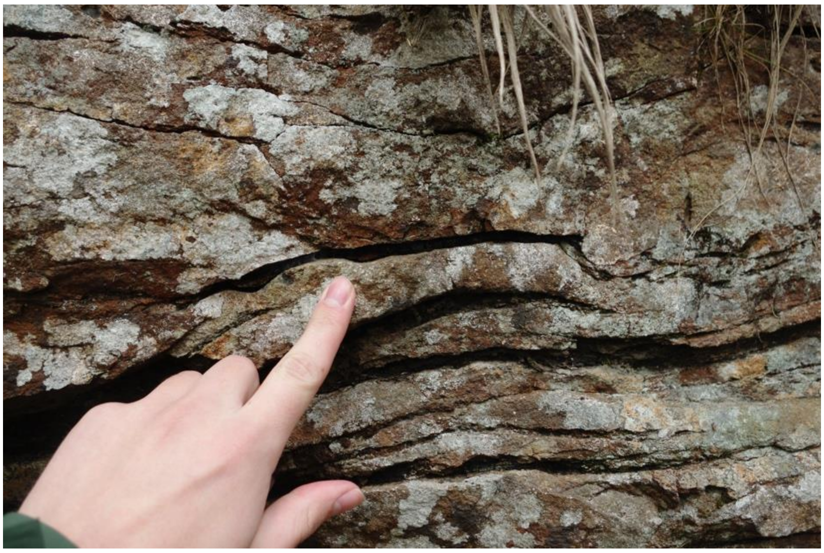

Supplementary Figure S2.2. Compressed, fragmented, macerated, coalified plant material at Loc2.2

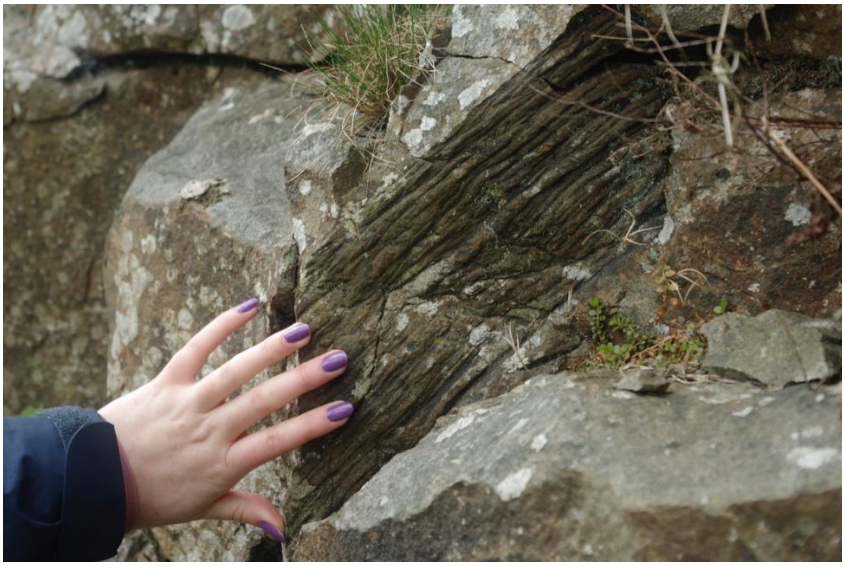

Supplementary Figure S2.3. *Lepidodendron* woody debris fossil at Loc. 2.3

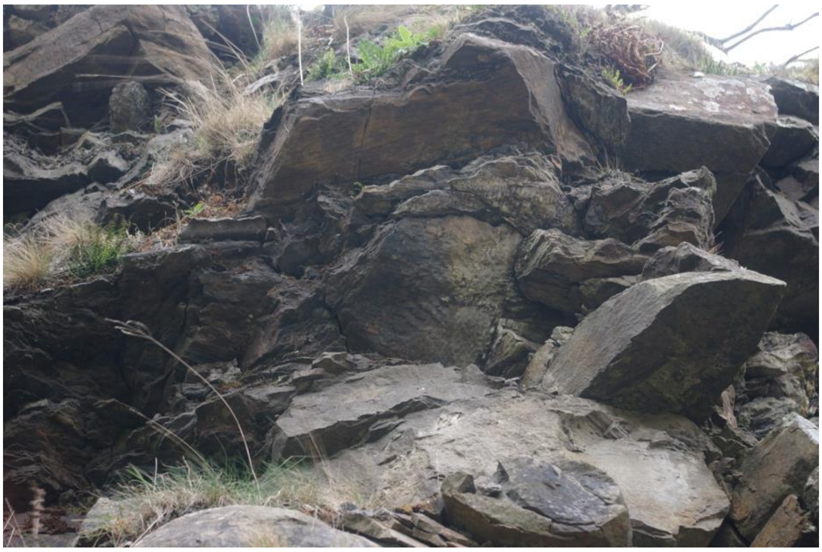

Supplementary Figure S2.4. *Lepidodendron* fossil in *conglomerate lithofacies* at Loc4.1

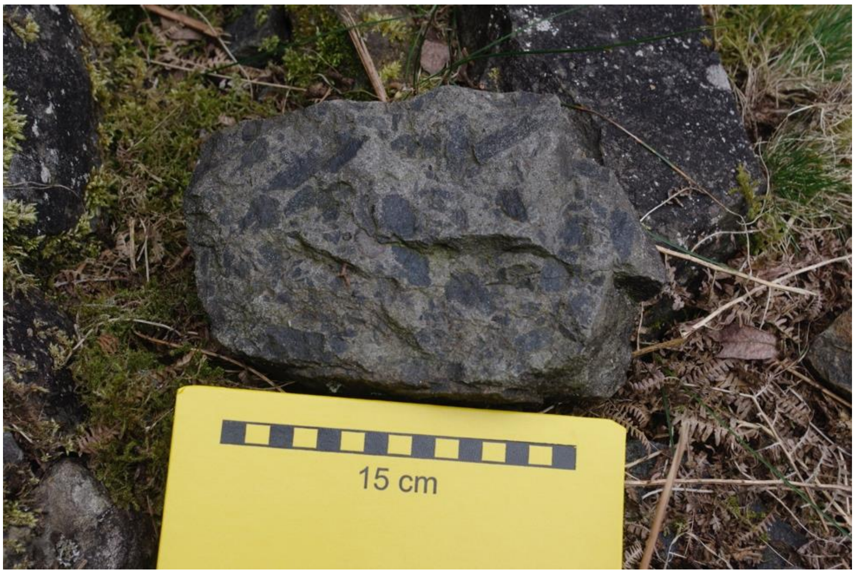

Supplementary Figure S2.5. Organic-rich fossilised plant matter at Loc4.1

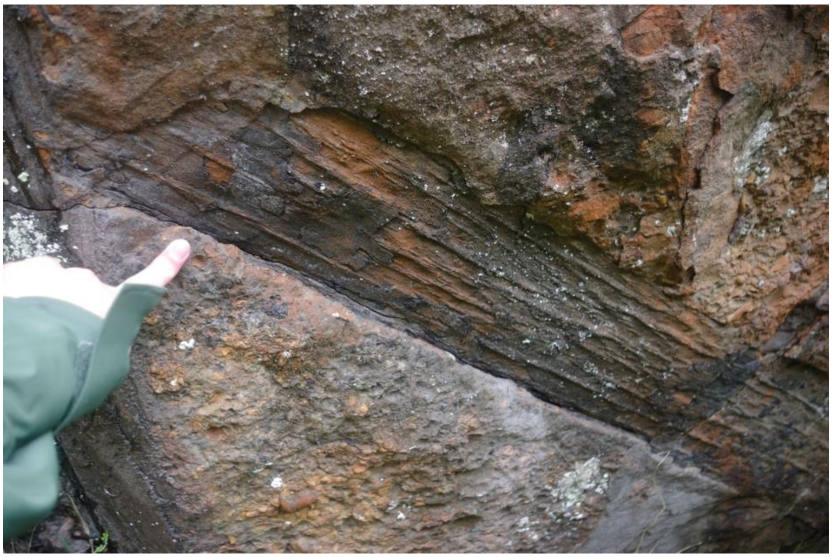

Supplementary Figure S2.6. Fossilised log of *Lepidodendron* in *conglomerate lithofacies* at Loc3.3.

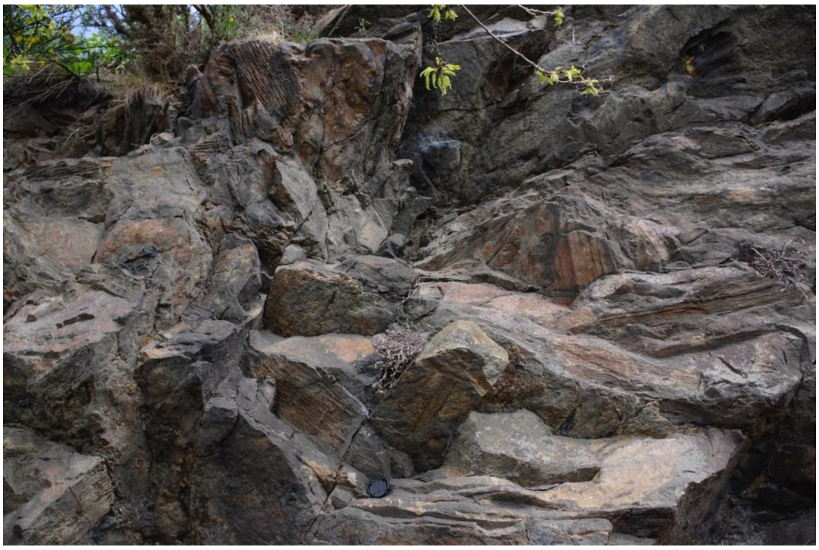

Supplementary Figure S2.7. Upwards view of conglomeratic unit, interpreted as log-jam deposit at Loc3.3, containing ~3 m of stacked woody debris fossils.

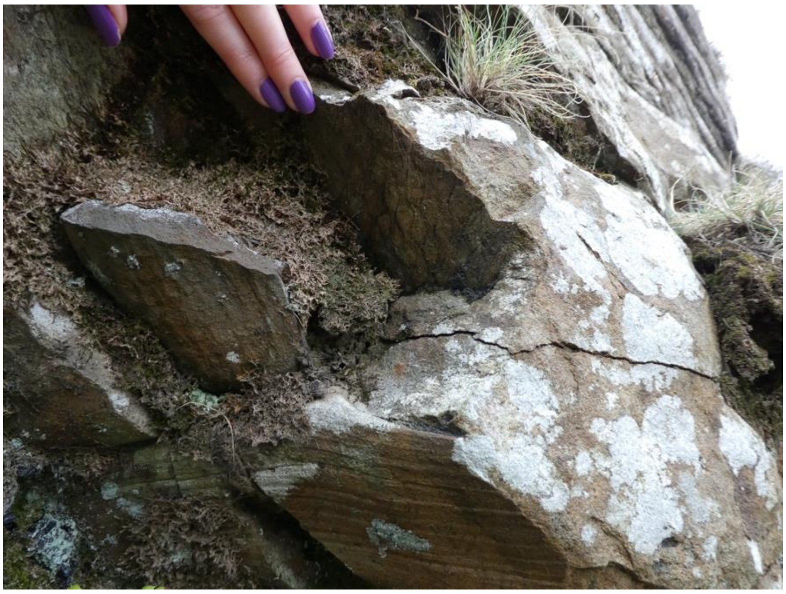

Supplementary Figure S2.8. Well-preserved leaf cushions on a *Lepidodendron* fossil in the *conglomerate lithofacies* at Loc2.1.

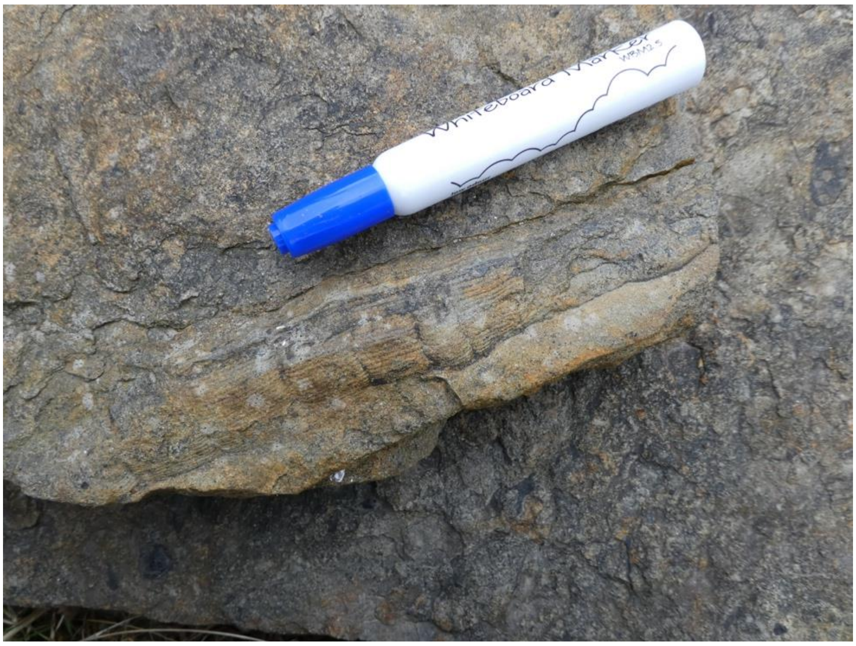

Supplementary Figure S2.9. Fossil cast of *Calamites* in a matrix of highly macerated vegetation and sand at Loc2.1

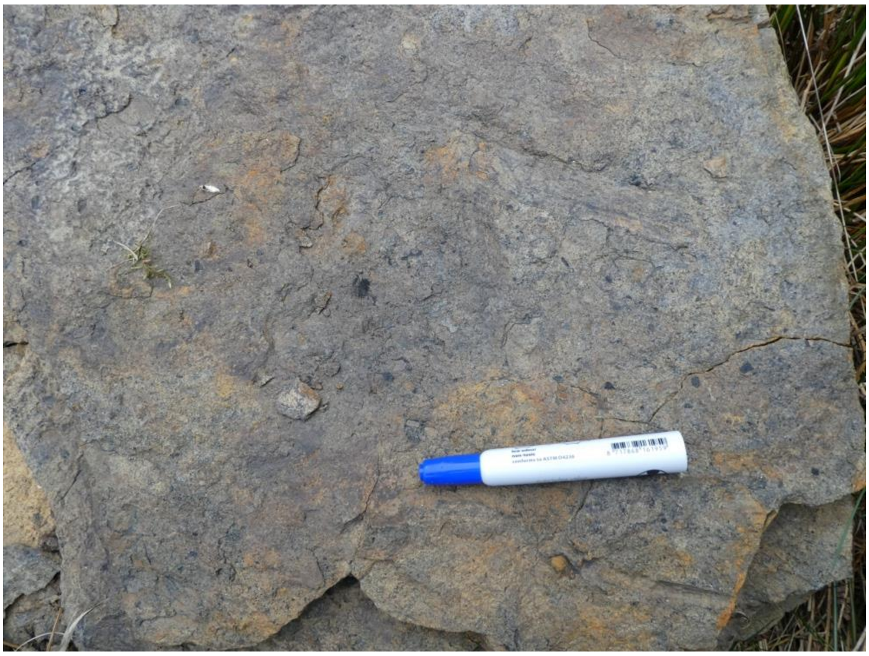

Supplementary Figure S2.10. Debris accumulation including *Calamites* fossil and a matrix of macerated vegetation and sand at Loc2.1

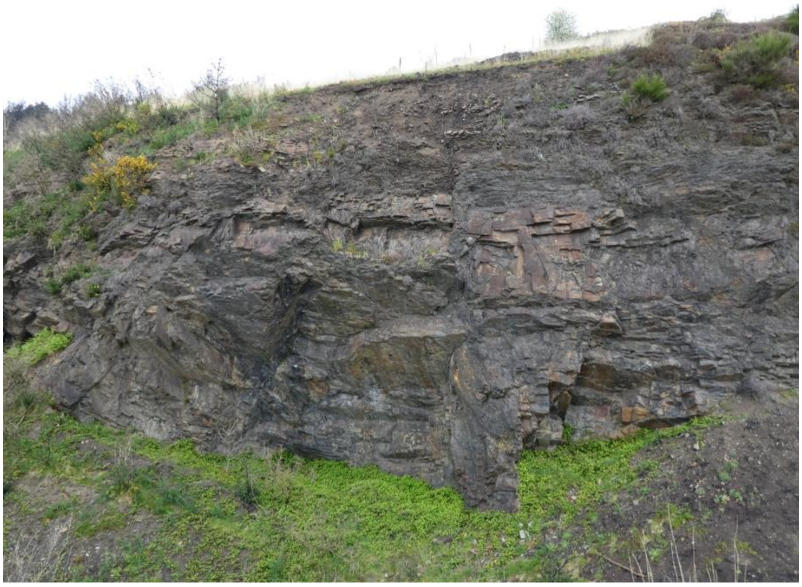

Supplementary Figure S2.11. Architectural view of Kilvey Hill (Loc3.3)

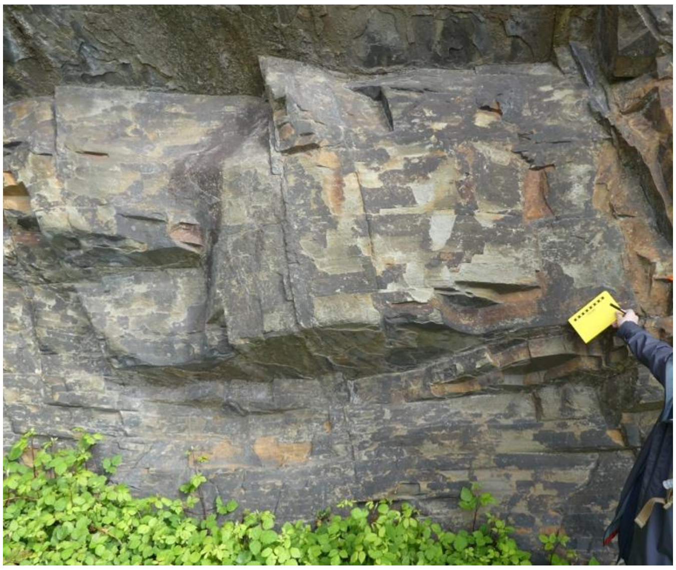

Supplementary Figure S2.12. Upper plane-bed lamination in sandstone at Loc3.4, found in association with log-jam deposits.

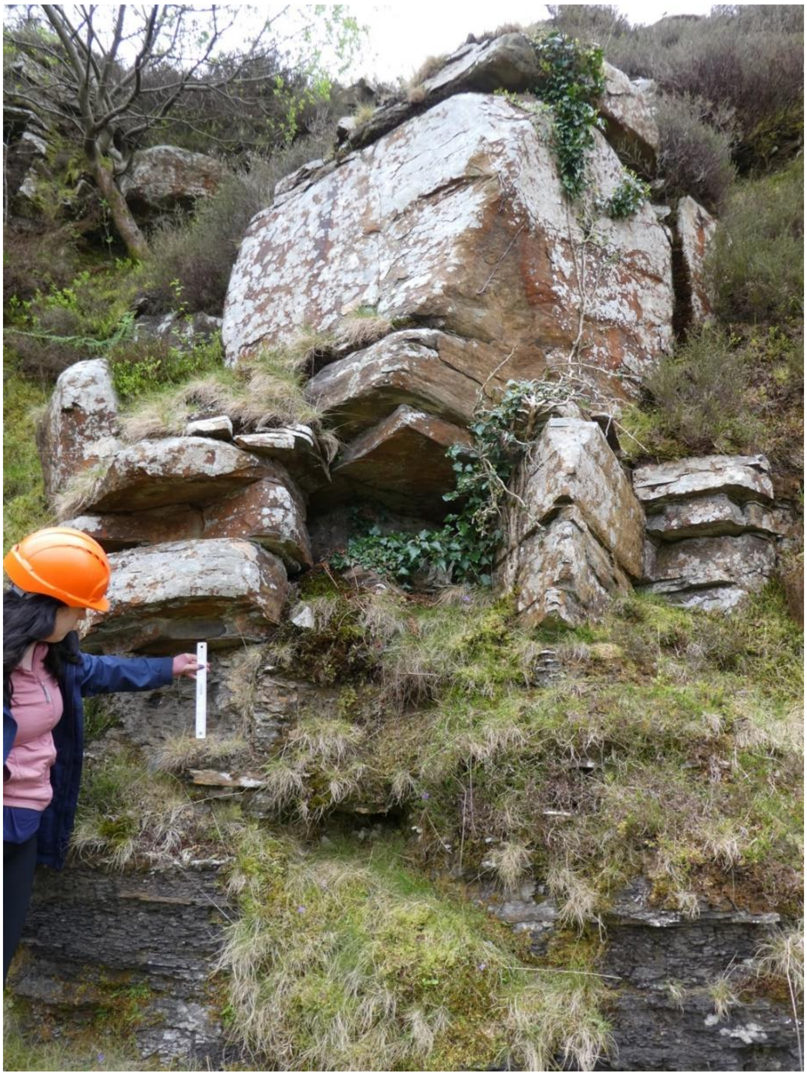

Supplementary Figure S2.13. Section of overbank deposits logged at Loc4.3

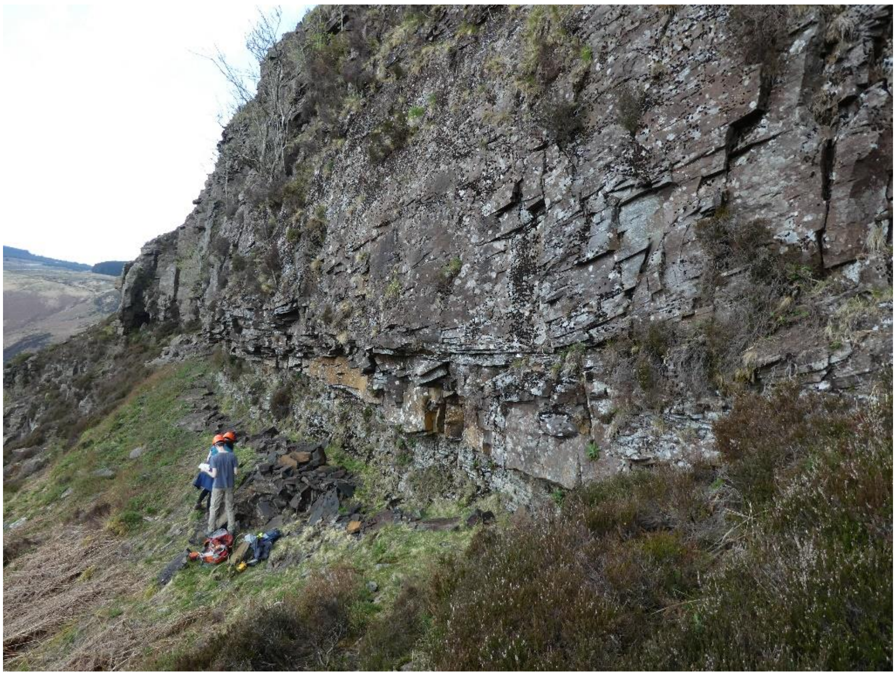

Supplementary Figure S2.14. Base of lateral accretion set at Loc7.3, where woody debris was documented in the *sandstone lithofacies.*

References

1. Leclair, S. F. & Bridge, J. S. Quantitative interpretation of sedimentary structures formed by river dunes. *Journal of Sedimentary Research* **71**, 713–716 (2001).
2. Bridge, J. & Bestt, J. Preservation of planar laminae due to migration of low-relief bed waves over aggrading upper-stage plane beds: comparison of experimental data with theory. *Sedimentology* **44**, 253–262 (1997).
3. Leclair, S. F., Bridge, J. S. & Wang, F. Preservation of Cross-strata Due to Migration of Subaqueous Dunes Over Aggrading and Non-aggrading Beds: Comparison of Experimental Data with Theory. *Geoscience Canada* (1997).
4. Paola & Borgman. *Reconstructing random topography from preserved stratification*. vol. 38 (1991).
5. Allen, J. R. L. Time-lag of dunes in unsteady flows: An analysis of nasner's data from the R. Weser, Germany. *Sediment Geol* **15**, 309–321 (1976).
6. Allen, J. R. L. Reaction, relaxation and lag in natural sedimentary systems: General principles, examples and lessons. *Earth Science Reviews* **10**, 263–342 (1974).
7. Martin, R. L. & Jerolmack, D. J. Origin of hysteresis in bed form response to unsteady flows. *Water Resour Res* **49**, 1314–1333 (2013).
8. Myrow, P. M., Jerolmack, D. J. & Perron, J. T. Bedform disequilibrium. *Journal of Sedimentary Research* **88**, 1096–1113 (2018).
9. Serinaldi, F., Loecker, F., Kilsby, C. G. & Bast, H. Flood propagation and duration in large river basins: a data-driven analysis for reinsurance purposes. *Natural Hazards* **94**, 71–92 (2018).
10. Musolino, G. & Ahmadian, R. A new approach in the design of evacuation/access routes using a fully conservative 2D model. in *38th IAHR World Congress - 'Water: Connecting the World'* vol. 38 4144–4155 (The International Association for Hydro-Environment Engineering and Research, 2019).
11. Nittrouer, J. A., Allison, M. A. & Campanella, R. Bedform transport rates for the lowermost Mississippi River. *J Geophys Res Earth Surf* **113**, (2008).
12. Leary, K. & Buscombe, D. Estimating Sand Bedload in Rivers by Tracking Dunes: a comparison of methods based on bed elevation time-series. *Earth Surface Dynamics Discussions* **8**, 161–172 (2019).
13. Sukhodolov, A. N., Fedele, J. J. & Rhoads, B. L. Structure of flow over alluvial bedforms: An experiment on linking field and laboratory methods. *Earth Surf Process Landf* **31**, 1292–1310 (2006).
14. Almeida, R. P. de *et al.* Large barchanoid dunes in the Amazon River and the rock record: Implications for interpreting large river systems. *Earth Planet Sci Lett* **454**, 92–102 (2016).
15. Bradley, R. W. & Venditti, J. G. Reevaluating dune scaling relations. *Earth Sci Rev* **165**, 356–376 (2017).
16. Wentworth, C. K. A Scale of Grade and Class Terms for Clastic Sediments. *https://doi.org/10.1086/622910* **30**, 377–392 (1922).

17. Trampush, S. M., Huzurbazar, S. & McElroy, B. Empirical assessment of theory for bankfull characteristics of alluvial channels. *Water Resour Res* **50**, 9211–9220 (2014).
18. Mahon, R. C. & McElroy, B. Indirect estimation of bedload flux from modern sand-bed rivers and ancient fluvial strata. *Geology* **46**, 579–582 (2018).
19. Greenberg, E., Ganti, V. & Hajek, E. Quantifying bankfull flow width using preserved bar clinoforms from fluvial strata. *Geology* **49**, 1038–1043 (2021).
20. Parker, G. On the cause and characteristic scales of meandering and braiding in rivers. *J. Fluid Mech* **76**, 457–480 (1976).
21. Lyster, S. J., Whittaker, A. C. & Hajek, E. A. *The problem of paleo-planforms*. *Earth and Planetary Science Letters* (2022).
22. Lyster, S. J., Whittaker, A. C., Hajek, E. A. & Ganti, V. Field evidence for disequilibrium dynamics in preserved fluvial cross-strata: A record of discharge variability or morphodynamic hierarchy? *Earth Planet Sci Lett* **579**, (2022).
23. Hayden, A. T., Lamb, M. P. & McElroy, B. J. Constraining the Timespan of Fluvial Activity From the Intermittency of Sediment Transport on Earth and Mars. *Geophys Res Lett* **48**, (2021).
24. Peyser, C. E. & Poulsen, C. J. Controls on Permo-Carboniferous precipitation over tropical Pangaea: A GCM sensitivity study. *Palaeogeogr Palaeoclimatol Palaeoecol* **268**, 181–192 (2008).
25. Tabor, N. J. & Poulsen, C. J. Palaeoclimate across the Late Pennsylvanian-Early Permian tropical palaeolatitudes: A review of climate indicators, their distribution, and relation to palaeophysiographic climate factors. *Palaeogeogr Palaeoclimatol Palaeoecol* **268**, 293–310 (2008).
26. Wood, J., McLeod, J. S., Lyster, S. J. & Whittaker, A. C. Rivers of the Variscan Foreland: fluvial morphodynamics in the Pennant Formation of South Wales, UK. *Journal of the Geological Society* (2022) doi:https://doi.org/10.31223/X5TK94.
27. Gupta, P. K., Chauhan, S. & Oza, M. P. Modelling surface run-off and trends analysis over India. *J. Earth Syst. Sci.* **125**, 1089–1102 (2016).
28. Jones, C. M. The sedimentology of Carboniferous fluvial and deltaic sequences; the Roaches Grit Group of the South-West Pennines and the Pennant Sandstone of the Rhondda Valleys. (Keele University, 1977).
29. Jones, J. A. & Hartley, A. J. Reservoir characteristics of a braid-plain depositional system: the Upper Carboniferous Pennant Sandstone of South Wales. *Geological Society Special Publications* **73**, 143–156 (1993).
30. Evans, B. G. An introduction to the South Wales Pennant Formation: its origin, outcrop and conservation. *Geologica Balcanica* **34**, 29–37 (2004).
31. Falcon-Lang, H. J., Cleal, C. J., Pendleton, J. L. & Wellman, C. H. Pennsylvanian (mid/late Bolsovian-Asturian) permineralised plant assemblages of the Pennant Sandstone Formation of southern Britain: Systematics and palaeoecology. *Rev Palaeobot Palynol* **173**, 23–45 (2012).
32. Burgess, P. M. & Gayer, R. A. Late Carboniferous tectonic subsidence in South Wales: implications for Variscan basin evolution and tectonic history in SW Britain. *J Geol Soc London* **157**, 93–104 (2000).
33. Kelling, G. Patterns of sedimentation in Rhondda Beds of South Wales. *The American Association of Petroleum Geologists Bulletin* (1968).

34. Howell, A. & Cox, A. H. On a group of red measures or coloured strata in the east Glamorgan and Monmouthshire coalfield. *South Wales Inst. Eng. Proc.* **43**, 139–174 (1924).
35. Moore, L. R. The sequence and structure of the southern portion of the East Crop of the South Wales Coalfield. *Geol. Soc. London Quart. Jour.* **103**, 261–300 (1948).
36. Moore, L. R. The geological sequence of the South Wales coalfield: the 'South Crop' and Caerphilly basin and its correlation with the Taff Valley sequence. *South Wales Inst. Eng. Proc.* **60**, 141–152 (1945).
37. Blundell, C. R. K. The succession and structure of the north eastern area of the South Wales coalfield. *Geol. Soc. London Quart. Jour.* **107**, 307–333 (1952).
38. Kelling, G. Sediment transport in part of the lower Pennant measures of South Wales. *L.M.J.U. Van Straaten, ed., Deltaic and shallow marine deposits* 177–184 (1964).
39. Jones, J. A. The influence of contemporaneous tectonic activity on Westphalian sedimentation in the South Wales Coalfield. *Devonian and Carboniferous Tectonics and sedimentation* **6**, 243–253 (1989).
40. Cleal, C. J. & Thomas, B. M. *Plant fossils of the British Coal Measures*. (The Palaeontological Association, 1994).
41. Gibling, M. R., Bashforth, A. R., Falcon-Lang, H. J., Allen, J. P. & Fielding, C. R. Log jams and flood sediment buildup caused channel abandonment and avulsion in the pennsylvanian of atlantic Canada. *Journal of Sedimentary Research* **80**, 268–287 (2010).
42. Thomas, B. A. & Cleal, C. J. Arborescent lycophyte growth in the late Carboniferous coal swamps. *Source: The New Phytologist* **218**, 885–890 (2018).
43. Dimichele, W. A., Pfefferkorn, H. W. & Gastaldo, R. A. Response of late Carboniferous and early Permina plant communities to climate change (2001).
44. Fielding, C. R., Allen, J. P., Alexander, J. & Gibling, M. G. Facies model for fluvial systems in the seasonal tropics and subtropics. *Geology* **37**, 623–626 (2009).
45. Davies, N. S. & Gibling, M. R. The sedimentary record of Carboniferous rivers: Continuing influence of land plant evolution on alluvial processes and Palaeozoic ecosystems. *Earth Sci Rev* **120**, 40–79 (2013).
46. Alexander, J., Fielding, C. R. & Jenkins, G. Plant-material deposition in the tropical Burdekin River, Australia: implications for ancient fluvial sediments. *Palaeogeogr Palaeoclimatol Palaeoecol* **153**, 105–125 (1999).
47. Fielding, C. R., Alexander, J. & Allen, J. P. The role of discharge variability in the formation and preservation of alluvial sediment bodies. *Sediment Geol* **365**, 1–20 (2018).
48. Trümper, S. *et al.* Late Palaeozoic red beds elucidate fluvial architectures preserving large woody debris in the seasonal tropics of central Pangaea. *Sedimentology* **67**, 1973–2012 (2020).
49. Falcon-Lang, H. J. & Bashforth, A. R. Pennsylvania uplands were forested by giant cordaitalean trees. *Geology* **32**, 417–420 (2004).
50. Falcon-Lang, H. J. & Bashforth, A. R. Morphology, anatomy, and upland ecology of large cordaitalean trees from the Middle Pennsylvanian of. *Rev Palaeobot Palynol* **135 (3–4)**, 223–243 (2005).
51. Bashforth, A. R. (Arden R., Canadian Society of Petroleum Geologists. & Geological Association of Canada. Late Carboniferous (Bolsovian) macroflora from the Barachois Group, Bay St. George Basin, southwestern Newfoundland, Canada. 123 (2005).

52. Ferguson, D. K. The origin of leaf-assemblages - new light on an old problem. *Rev Palaeobot Palynol* **46**, 117–188 (1985).
53. Melrose, C. S. A. Fossilized Forests of the Lower Carboniferous Horton Blufff Formation, Nova Scotia. (2003).
54. Wagner, R. H. & Diez, J. B. Verdeña (Spain): Life and death of a Carboniferous forest community. *C R Palevol* **6**, 495–504 (2007).
55. Hagerty, D. J., Spoor, M. F. & Ullrich, C. R. Bank failure and erosion on the Ohio river. *Eng Geol* **17**, 141–158 (1981).
56. Thorne, C. R., Russell, A. P. G. & Alam, M. K. Planform pattern and channel evolution of the Brahmaputra River, Bangladesh. *Geological Society, London, Special Publications* **75**, 257–276 (1993).
57. Coleman, J. M. Brahmaputra river: Channel processes and sedimentation. *Sediment Geol* **3**, 129–239 (1969).
58. Martin, C. A. L. & Turner, B. R. Origins of massive-type sandstones in braided river systems. *Earth Science Reviews* **44**, 15–38 (1998).
59. Stewart, W. N. (Wilson N. & Rothwell, G. W. Paleobotany and the evolution of plants. 521 (2009).
60. Phillips, T. L. & Dimichele, W. A. Comparative Ecology and Life-History Biology of Arborescent Lycopsids in Late Carboniferous Swamps of Euramerica. *Annals of the Missouri Botanical Garden* **79**, 560–588 (1992).
61. Alexander, J., Fielding, C. R. & Jenkins, G. Plant-material deposition in the tropical Burdekin River, Australia: implications for ancient fluvial sediments. *Palaeogeogr Palaeoclimatol Palaeoecol* **153**, 105–125 (1999).
62. Robison, E. G. & Beschta, R. L. Characteristics of coarse woody debris for several coastal streams of southeast Alaska, USA. *Canadian Journal of Fisheries and Aquatic Sciences* **47**, 1684–1693 (1990).
63. Burns, C. E., Mountney, N. P., Hodgson, D. M. & Colombera, L. Stratigraphic architecture and hierarchy of fluvial overbank splay deposits. *J Geol Soc London* **176**, 629–649 (2019).
64. Galloway, W. E. & Hobday, D. K. Terrigenous Clastic Depositional Systems: Applications to Fossil Fuel and Groundwater Resources: Applications to Petroleum, Coal, and Uranium Exploration by Galloway, William E., Hobday, David K.: (1996) | medimops. (Springer, 1996).
65. Arnaud-Fassetta, G. Dyke breaching and crevasse-splay sedimentary sequences of the rhône delta, France, caused by extreme river-flood of december 2003. *Geografia Fisica e Dinamica Quaternaria* **36**, 7–26 (2013).
