# Supplementary material for: Quantitative constraints on flood variability in the rock record
Source: Nat Commun. 2023 Jun 8;14:3362. doi: 10.1038/s41467-023-38967-8 (PMC10250471; doi:10.1038/s41467-023-38967-8)
Supplement: Supplementary file 2 — Description of Additional Supplementary Files [file 41467_2023_38967_MOESM2_ESM.pdf]

Description of Additional Supplementary Files

File Name: Supplementary Data 1

Description: **Localities and access (.kmz)**

A Google Earth .kmz file showing each locality, represented stratigraphy, and access information.

File Name: Supplementary Data 2

Description: **Primary field data and statistical analyses (.xlsx)**

An excel workbook with all collected primary field data and statistical tests.

- S2a: Data log outlining collected datasets
- S2b: Cross-set height distributions
- S2c: Maximum cross-set height measurements
- S2d: Accretion and bedding measurements
- S2e: Package thickness measurements
- S2f: Woody debris measurements
- S2g: *CV* of cross-sets associated with woody debris
- S2h: Statistical test on cross-set heights between members
- S2i: Statistical test on *CV* between members
- S2j: Statistical test on *CV* between debris-associated and non-debris-associated cross-sets
